# Production of GcMAF with Anti-Inflammatory Properties and Its Effect on Models of Induced Arthritis in Mice and Cystitis in Rats

**DOI:** 10.3390/cimb46100650

**Published:** 2024-09-28

**Authors:** Svetlana S. Kirikovich, Evgeniy V. Levites, Anastasia S. Proskurina, Genrikh S. Ritter, Evgeniya V. Dolgova, Vera S. Ruzanova, Sofya G. Oshihmina, Julia S. Snegireva, Svetlana G. Gamaley, Galina M. Sysoeva, Elena D. Danilenko, Oleg S. Taranov, Alexandr A. Ostanin, Elena R. Chernykh, Nikolay A. Kolchanov, Sergey S. Bogachev

**Affiliations:** 1 Institute of Cytology and Genetics of the Siberian Branch of the Russian Academy of Sciences, 630090 Novosibirsk, Russia; levites@bionet.nsc.ru (E.V.L.); asproskurina@gmail.com (A.S.P.); ritter@bionet.nsc.ru (G.S.R.); dolgova.ev@mail.ru (E.V.D.); ruzanova@bionet.nsc.ru (V.S.R.); s.oshikhmina@g.nsu.ru (S.G.O.); kol@bionet.nsc.ru (N.A.K.); 2 Faculty of Biotechnologies, ITMO University, 191002 Saint Petersburg, Russia; snegireva_julia@mail.ru; 3 State Research Center of Virology and Biotechnology “Vector”, 630559 Koltsovo, Russia; gamaley_sg@vector.nsc.ru (S.G.G.); kanz_imbt@vector.nsc.ru (G.M.S.); danilenko_ed@vector.nsc.ru (E.D.D.); taranov@vector.nsc.ru (O.S.T.); 4 Research Institute of Fundamental and Clinical Immunology, 630099 Novosibirsk, Russia; ostanin62@mail.ru (A.A.O.); ct_lab@mail.ru (E.R.C.)

**Keywords:** DBP, sialic acid/galactose residue deglycosylation, GcMAF, CLEC10A, M1/M2 macrophages, IL-1β, TNF-α, TGF-β, IL-10, induced cystitis, induced arthritis

## Abstract

Vitamin D_3_ transporter (DBP) is a multifunctional protein. Site-specific deglycosylation results in its conversion to group-specific component protein-derived macrophage activating factor (GcMAF), which is capable of activating macrophages. It has been shown that depending on precursor conversion conditions, the resulting GcMAF activates mouse peritoneal macrophages towards synthesis of either pro- (IL-1β, TNF-α—M1 phenotype) or anti-inflammatory (TGF-β, IL-10—M2 phenotype) cytokines. The condition for the transition of the direction of the inflammatory response of macrophages when exposed to GcMAF is the initial glycosylated state of the population of DBP molecules and the associated effective deglycosylation of DBP by β-galactosidase. In vivo experiments with GcMAF exhibiting anti-inflammatory properties on models of induced arthritis in mice and cystitis in rats indicate a significant anti-inflammatory effect of the macrophage activator. The feasibility of unidirectional induction of anti-inflammatory properties of macrophages allows creation of combined therapeutic platforms where M2 macrophages are among the key therapeutic components.

## 1. Introduction

Macrophage activation is a process involving two components: the macrophage-activating factor per se and its target (in this case, macrophages of different localization: peritoneal macrophages, Kupffer cells, alveolar macrophages, and microglia).

GcMAF is one of specific macrophage-activating factors belonging to the group of plasma α-globulins [1]. Literature analysis reveals a clear trend, with a major focus placed on characterization of different properties of GcMAF precursor (vitamin D_3_–binding protein, DBP) and MAF, and primarily on the analysis of the protein component that is responsible for the activation event [2,3,4].

In the analyzed studies, assessment of phagocytic activity of macrophages and efficiency of NO synthesis are considered a major and commonly used indicator of macrophage activation [5,6,7,8]. Numerous studies demonstrate that macrophage activation is associated with the presence of a monosaccharide composite within a vitamin D_3_–binding protein molecule.

In 1983, Viau et al. [9] were first to show that vitamin D_3_ transporter involves a trisaccharide composed of galactose, sialic acid, and GalNAc moiety covalently bound to Thr residue of the protein via an O–glycosidic bond. They also described a trisaccharide variant carrying two sialic acid residues.

The intense research into the precursor as a macrophage-activating factor was started with studies conducted by the team led by N. Yamamoto, who demonstrated that inflamed T and B cells are involved in conversion of DBP to a macrophage-activating factor and that the event of trisaccharide deglycosylation by membrane-bound enzymes, β–galactosidase residing on B cells and sialidase residing on T cells, is the pivotal event in conversion [7,10,11,12,13]. The main problem researchers faced in further studies was “what should be considered a macrophage-activating factor GcMAF”.

The human Gc system comprises over 120 alleles in the human population; three of these alleles (Gc2, Gc1F, and Gc1S) are dominant [14,15] and can exist in both homo- and heterozygous states. The Gc protein contains three major sugar residues. Studies have demonstrated that different allelic forms of the protein undergo glycosylation in different ways. Thus, Nagasawa et al. [8] conducted systematic evaluation of glycosylation of the macrophage-activating factor and its precursor. They found that Gc1F can carry a branched trisaccharide: GalNAc, galactose and a sialic acid residue, where GalNAc is attached to Thr418 and Thr420 residues of the core protein via an O–glycosidic bond [16]. In Gc1S, the sialic acid residue is substituted with a mannose residue. It was shown that Gc2 carries a GalNAc: galactose disaccharide associated with Thr418 via a GalNAc moiety; however, most of the protein in this allelic form is nonglycosylated [16]. Further studies have complemented this systematization. It was shown that GcS1 can also carry the GalNAc–galactose disaccharide [17], and the detected differences in the type of saccharide residues (galactose vs. mannose) could mean that posttranslational glycosylation is nonstoichiometric [17].

An alternative structure of glycosylation sites consisting of linearly connected saccharides (the sialic acid–galactose–GalNAc residue) was proposed for Gc1F and Gc1S [8,18,19]. Uto et al. suggested that the residue sequence for Gc1F could also be galactose–sialic acid–GalNAc–Thr and that the phagocytic activity of peritoneal macrophages (PMs) is activated by a protein hydrolyzed by both β–galactosidase and sialidase [6]. In another study, it was demonstrated by ESI–MS that the trisaccharide bound to a precursor molecule by O–glycosidic bond and consisting of a sialic acid residue, galactose and a GalNAc moiety is detected for both Gc1F and Gc1S forms and is composed of linearly arranged saccharides [20,21]. Galactose–GalNAc–Thr, rather than the GalNAc monosaccharide as previously suggested by other researchers, is the main O–glycan in GcMAF obtained from the Gc1F allele product [8]. No mannose residues in any form were detected in this work. An important finding was that β–galactosidase used in different studies is characterized by different abilities to affect (hydrolyze) the galactose residue. Meanwhile, actual galactose elimination by a specific enzyme derived from bovine testes does not functionally convert DBP to GcMAF. Borges and Rehder [22] suggested that the enzyme isolated from *E. coli*, which is employed in most studies, does not eliminate galactose from the precursor but may convert DBP by inverting lactose to allolactose or otherwise modifying the monosaccharide [22].

Furthermore, based on the ESI–MS analysis of glycosylation degree of plasma DBP in patients with several cancer types, Rehder et al. hypothesized that GalNAc monosaccharide is bound to Thr420, the site where the trisaccharide is primarily located, which is consistent with numerous findings reported by other researchers [23]. They also believe that there exists an allelic form of Gc1 that can be glycosylated by a disaccharide at 418Thr position and that this very derivative is the actual GcMAF [23].

In their recent study, Kanie et al. [24] conducted MS and EMRS analyses and revealed that the analyzed protein contains two glycosylation sites carrying the same saccharide residues. It is known that pH variation can alter the isomeric structure of saccharide residues linked by an O–glycosidic bond, carrying proline in the Thr418-Pro419-Thr420 sequence. It was hypothesized that the glycopeptide carrying GalNAc at Thr420 and Thr418 positions may also exist as *cis*- and *trans*-isomers associated with Pro419. These findings indicate that the structure of the Gc protein trisaccharide is a linear sequence of saccharide residues (sialic acid–galactosidase and GalNAc). This structure can be linked to Thr420 or Thr418 via an O–glycosidic bond, but only to one of these residues within the same molecule [24].

Apparently, it cannot be clearly inferred from the data obtained by analyzing isolated purified variants of the protein, which form of the molecule actually GcMAF is, since different cognate representatives of the GcMAF candidate are analyzed by different approaches and in different cases. Therefore, there arise different, often conflicting, ideas about the nature of protein’s properties, making the experimental search utterly confusing.

The available experimental data suggest that the macrophage-activating factor is a protein molecule carrying either a fully deglycosylated GalNAc moiety or a GalNAc–galactose disaccharide (a modified sugar residue). Both sugar residue variants can be formed by simultaneous enzymatic treatment of either allelic form of the precursor. This means that multiple variants of molecules can exist simultaneously and that it is impossible to isolate a single variant of glycosylated molecule. If we add the following aspects—(1) the population-level multiplicity of protein alleles; (2) the fact that they can form heterozygotes; (3) the lack of information about whether these alleles can also be converted to the macrophage-activating factor; and (4) differences in activities of the enzymes used by different research teams—then identifying a single type of molecule characterized by a particular glycosylation variant, which actually is GcMAF, will always be an uncertainty that cannot be avoided.

The central objective of this study was to search for conditions that allow targeted conversion of vitamin D_3_ binding protein precursor (DBP) to GcMAF exhibiting marked anti-inflammatory characteristics, which will enable programmed induction of the M2 phenotype of macrophage-enforcing innate immunity. Furthermore, using the GcMAF preparation exhibiting anti-inflammatory properties, we assessed its anti-inflammatory effects in the mouse model of induced arthritis and the rat model of interstitial cystitis (IC). Utilization of these experimental models of diseases that are common in humans allow one to assess the breadth of the therapeutic potential of GcMAF exhibiting anti-inflammatory properties.

## 2. Materials and Methods

### 2.1. Experimental Design

Macrophages are functionally plastic immune surveillance cells having multiple functions [25]. The available studies, starting with the earliest ones, focused exclusively on their phagocytic activity and induction of NO synthesis, which are based on the interaction between GcMAF and the macrophage Fc receptor. Nonetheless, it is known that macrophages can have a number of other functions depending on their functional status, primarily eliciting the pro- and anti-inflammatory response. At its simplest, those are the three major phenotypes of M1, M0, and M2 macrophages. We found a single paper discussing that macrophages are multifunctional cells and that their different properties can be activated [26].

In our recent studies, by assessing the efficiency of mRNA expression by two inflammatory mediators TNF-α and IL-1β, as well as two major anti-inflammatory cytokines TGF-β and IL-10, we have demonstrated that GcMAF modulates the pro/anti-inflammatory activity of PMs [27,28,29]. Synthesis of the analyzed cytokines was found to be dependent on interaction between GcMAF and CLEC10A, the specific carbohydrate-binding macrophage receptor. Binding between a deglycosylated DBP molecule (GcMAF) and three derivatives of the C-type lectin receptor CLEC10A residing on the plasma membrane of phagocytes was shown to be responsible for a specific direction of immune response in PMs. Supplementary Figure S1 shows graphical representation of the structural (core) elements used to visualize the interaction between different glycosylated forms of DBP/GcMAF and the CLEC10A complex receptor.

It turned out that during the GcMAF/CLEC10A interaction, synthesis of specific cytokines is induced if a complex aggregate is formed as a result of double bonding between a ligand (GcMAF) and a 29 kDa CLEC10A derivative as well as one of the two CLEC10A derivatives (the 63 kDa or 65 kDa one) carrying Ca^2+^ molecules, which is internalized into the cytoplasm as a component of acidified endosomes [28]. The details of this process can be characterized as follows. GcMAF forms a complex with a 29 kDa anchor derivative CLEC10A on PMs via the core molecular backbone. GcMAF associated with the 29 kDa derivative further contacts one of the two high-molecular-weight derivatives of 63 kDa or 65 kDa CLEC10A via the GalNAc glycosidic residue. If a second binding between the complex receptor and GalNAc occurs for the 63 kDa high-molecular-weight derivative on PMs, it is accompanied by eliciting of anti-inflammatory responses (TGF-β and IL-10 mRNA synthesis), and we denote such GcMAF as anti-GcMAF. If a second binding between the complex receptor and GalNAc occurs for the 65 kDa high-molecular-weight derivative on PMs, it is accompanied by eliciting proinflammatory responses (TNF-α and IL-1β mRNA synthesis), and such GcMAF is denoted as pro–GcMAF. If a second binding of the ligand to the 63/65 kDa CLEC10A derivatives does not take place (e.g., in the absence of Ca^2+^ ions), cytokine mRNA synthesis either does not occur or cytokine mRNA is synthesized chaotically, without any clear pattern (Supplementary Figure S2A–D) [28]. Moreover, synthesis of mRNA of cytokines under analysis is not induced if the ligand binds to the 63 kDa and 65 kDa derivatives on PMs, while not interacting with the 29 kDa derivative (e.g., GalNAc as a monopreparation) [28].

In our studies, we use pooled plasma collected from donors; it is a new composition for each series of experiments. DBP contained in donor plasma consists of molecules characterized by different glycosylation degrees (possibly at different sites on the molecule, at Tre418 or Tre420 position).

Based on the available data pool, there can be the following variants of glycosylation site: GalNAc, GalNAc + galactose, GalNAc + sialic acid, and GalNAc + galactose + sialic acid (in any form, either branched or sequentially arranged) (Supplementary Figure S2(A1)). The number of variants of molecules with different glycosylation degrees varies from patient to patient. The frequencies of different glycosylation variants also vary [7,30].

A question has arisen: how can consistent results be achieved under the aforementioned multifactorial uncertainty (namely, GcMAF preparation with desired characteristics that would guide the direction of synthesis of mRNA of either pro– (TNF-α and IL-1β) or anti-inflammatory (TGF-β and IL-10) cytokines)?

In this connection, when searching for the modes of producing GcMAF exhibiting either pro- or anti-inflammatory characteristics in the present study, we chose the approach involving exhaustive search of variants based on the final result (emergence of pro- or anti-inflammatory characteristics resulting from selective deglycosylation) without detailed analysis of molecular transformations of the analyzed protein (a mixture of cognate proteins): the “black box principle”. A system represented as a “black box” is considered to have some kind of “input” for data entry and “output” for displaying the results of the events that have occurred; it is unknown what processes occur during operation of the system. It is assumed that the state of outputs functionally depends on the state of inputs.

Let the entire set of possible candidates for functioning as GcMAF be viewed as a “black box”. By varying the conditions of DBP conversion (deglycosylation), we can find the mode where the final result is predictable and consistent, namely, mRNA synthesis of exclusively pro-inflammatory or exclusively anti-inflammatory cytokines, without analyzing the event within the uncertainty field. This very approach was used in the present study.

Nevertheless, by knowing many molecular characteristics of GcMAF obtained in our studies and taken from the literature, we attempted to illustrate the potential mechanistic schemes of interactions between different forms of DBP/GcMAF and CLEC10A leading to synthesis of mRNA of analyzed cytokines inside the “black box”.

The null hypothesis states that an equal number of molecules with all glycosylation variants are present in plasma: GalNAc, GalNAc + galactose, GalNAc + sialic acid, GalNAc + galactose + sialic acid (e.g., three molecules visualized in the figures for each variant, and a total of 12 schematic molecules (Supplementary Figure S2(A1)).

To search for regularities of the emergence of pro- or anti-inflammatory properties in the resulting GcMAF preparation, we visualized the initial pattern of compositions of the molecules with different variants of hinged sugar moieties that are present in pooled plasma collected from several donors based on the available data.

As mentioned above, the common GcMAF variant (the deglycosylated form with terminal GalNAc), which induces phagocytic activity and NO synthesis, is formed via complete detachment of galactose and sialic acid, while the terminal GalNAc moiety is preserved. We have previously shown that non-converted DBP from different donors can induce murine PMs towards low-level pro-inflammatory cytokine synthesis and induce phagocytic activity [28]. It implies that a certain insignificant portion of the mixture of DBP molecules was completely deglycosylated (galactose and sialic acid residues were detached). It also means that the glycosylated variants of the molecules were much more abundant compared to the completely deglycosylated variants of protein molecules. Therefore, we presented this variant of protein ratio in a visual format as a single visual molecule of the deglycosylated form carrying terminal GalNAc and ten molecules of the glycosylated protein variant (Supplementary Figure S2(A1)).

We also present the GalNAc + galactose variant as only one visual molecule (Supplementary Figure S2(A2)). The experimental data reported in the Results section argue in favor of this variant. It was shown in the experiment that in the presence of an excessive amount of β–galactosidase, the GcMAF preparation does not exhibit proinflammatory properties, meaning that the number of GalNAc + galactose molecules, which would provide the terminal GalNAc and impart proinflammatory characteristics during enzymatic treatment, in the initial substrate is insignificant and loses competitive binding to the receptor to another type of molecules. It is also consistent with the results of our studies demonstrating that DBP from different donors without using a conversion procedure can induce murine PMs toward synthesis of anti-inflammatory cytokines (Supplementary Figure S2E) [28].

In this scenario, in the selected visualization format (one GalNAc molecule, one GalNAc + galactose molecule, three GalNAc + sialic acid molecules, and three GalNAc + galactose + sialic acid molecules), different types of DBP molecules having different glycosylation patterns will bind either to the affine modalities to form double complexes or separately to the GalNAc epitope, bypassing binding to the 29 kDa anchor derivative, which depends on the number of protein molecules. Chaotic mRNA synthesis of analyzed cytokines will be observed, proceeding differently in different cases depending on the glycosylation pattern and the number of molecules with different patterns (Supplementary Figure S2A–C). An excessive amount of activated GcMAF causes competition between the “anchor” 29 kDa CLEC10A derivative and the high-molecular-weight 63/65 kDa CLEC10A derivatives. We assume that mRNA synthesis of the analyzed cytokines by PMs is completely inhibited if the “anchor” 29 kDa + 63/65 kDa complex cannot be formed (Supplementary Figure S2D). The engagement between GcMAF and the 29 kDa derivative is apparently labile, and the macrophage-activating factors persistently undergo association and dissociation. Supplementary Figure S2E shows the summarized experimental results of analyzing mRNA synthesis by PMs activated by different DBP variants harvested from four different donors [28], which illustrates the proposed mechanistic scheme for the ratio between DBP variants in plasma.

In Section 3.1. of this study, we attempted to use the approach described above to find a mode of DBP conversion that would predictably and consistently produce a GcMAF form exhibiting exclusively anti-inflammatory characteristics. We chose an approach involving enzymatic treatment of DBP in a solution under the conditions when glycosylation sites were naturally available for the enzyme. The logically consistent visualized schemes mechanistically depicting the different variants of GcMAF glycosylation and variants of their interaction with CLEC10A eliciting the pro-/anti-inflammatory are presented.

In Section 3.2 of the study, using the GcMAF preparation exhibiting anti-inflammatory properties, we assessed its anti-inflammatory effects in the mouse model of induced arthritis and the rat model of interstitial cystitis.

### 2.2. Experimental Animals

All the experiments involving animals were conducted in strict compliance with the principles of humanity in accordance with the European Community Council Directives (86/609/EEC) and approved by the Animal Care and Use Committee of the Institute of Cytology and Genetics SB RAS (Novosibirsk, Russia). Before and during the experiment, the animals were housed under standard vivarium conditions and fed a balanced diet.

Male 2- to 6-month-old C57BL/6 mice (weight, 18–24 g) were used to assess the effect of DBP and GcMAF on activation of peritoneal macrophages. The animals were kept in groups of 6–10 mice per cage with ad libitum access to food and water. Mice were sacrificed by cervical dislocation. 

Experiments on induction of adjuvant arthritis were conducted using 51 white outbred ICR mice (11 males and 40 females; body weight, 20–25 g) and 58 male C57BL/6 mice (body weight, 22–24 g). 

Cystitis was induced in 32 sexually mature male Wistar rats (initial body weight, 250–300 g). 

### 2.3. GcMAF Preparation

Vitamin D_3_–binding protein (DBP) was isolated from pooled human plasma collected from five donors by affinity chromatography on a 25-OH-D_3_/Sepharose^®^ column [27]. The next stage, namely, conversion of DBP to GcMAF, was carried out using two methods: either in solution or by a rapid method directly on a chromatographic column (“directly on resin”) [28].

ß-galactosidase (Sigma, St. Louis, MO, USA) and sialidase (Sigma, St. Louis, MO, USA) enzymes were added to DBP both simultaneously and individually to produce GcMAF in the solution. Thus, in order to produce GcMAF using both enzymes, equal doses (0.01 U/μg Gc) of ß-galactosidase (Sigma, St. Louis, MO, USA) and sialidase (Sigma, St. Louis, MO, USA) were added to the solution. In individual experiments, DBP was treated with different doses of ß-galactosidase alone (per μg Gc): 0.5 U, 0.1 U, 0.02 U, and 0.04 U. For GcMAF production using sialidase alone, DBP treatment was performed in the solution containing only sialidase (Sigma, St. Louis, MO, USA) at a dose of 0.01 U/μg Gc. The samples were incubated at 37 °C on a rocking shaker for 2 h. The reaction was terminated using ice, and the samples were refrigerated.

### 2.4. Ex Vivo Activation of PMs with DBP, GcMAF and Pure Enzymes

PMs were isolated from intact C57BL/6 mice and precipitated by centrifugation at 400× *g* for 7 min. The cells were resuspended in RPMI−1640; concentration was counted in a Goryaev’s chamber. PMs (1 × 10^6^ cells/well) were cultured in RPMI−1640 medium (BioloT, St. Petersburg, Russia) supplemented with 10% FBS (HyClone, Logan, UT, USA) and 40 μg/mL gentamicin in 24-well plates for 12 h. Next, the medium was replaced with RPMI−1640 in the absence (control) or presence of DBP or GcMAF. The macrophage-activating factors were added to each well at a dose of 1 µg.

For testing the effect of pure enzymes on macrophage activation, a solution of enzymes in PBS at a dose corresponding to 1 µg DBP was added to each well (10 mU β–galactosidase and sialidase per well). After activation, all the samples were incubated under absolutely identical conditions, and the effect observed was caused solely by MAF. The cells were incubated in an atmosphere of CO_2_ (Memmert, USA LLC, Eagle, WI, USA) at 37 °C for 3 h. After 3 h, the cells were washed to remove the MAF. The activated and control PMs were lysed with TRIzol Reagent (Thermo Fisher Scientific, Waltham, MA, USA) to obtain total RNA. Real-time PCR results were normalized to the level of mRNA isolated from PMs treated only with non-supplemented RPMI−1640.

### 2.5. Obtaining cDNA

Total RNA was isolated from peritoneal macrophages using TRIzol Reagent (Thermo Fisher Scientific, Waltham, MA, USA) in accordance with the manufacturer’s instructions. The amount of RNA was measured on a Qubit 4 fluorometer (Thermo Fisher Scientific, Waltham, MA, USA). Reverse transcription PCR was carried out on a poly-A mRNA template using a T100 Thermal Cycler amplifier (Bio-Rad Laboratories, Inc., Hercules, CA, USA) and an MMLV RT kit (Evrogen, Moscow, Russia) according to the manufacturer’s protocol.

### 2.6. Real-Time PCR

PCR analysis and the sequences of primers used in this study were reported in our previous publication [28]. Real-time PCR was carried out in 96-well plates using BioMaster HS-qPCR SYBR (2×) reaction mix (BIOLABMIX LLC, Novosibirsk, Russia) according to the manufacturer’s protocol on a QuantStudio5 PCR system (Thermo Fisher Scientific, Waltham, MA, USA). Real-time qPCR analysis of each sample was performed in three replicates. The relative expression level was determined using the 2^−ΔΔCt^ method. Intact nontreated PMs were used as the control group; the expression level of the target gene in them was assumed to be equal to 1. The GAPDH gene was used as reference. The cycling parameters for the TNF-α, IL-1β, TGF-β, and IL-10 genes were as follows: 95 °C for 10 min, 40 cycles of 95 °C for 30 s, 59 °C for 30 s, 72 °C for 30 s, with a final melting step involving slow heating from 6 to 95 °C.

### 2.7. Assessment of the Effect of Anti-GcMAF Preparation on the Development of Experimentally Induced Adjuvant Arthritis in Mice

Experimental adjuvant arthritis was induced by injecting 0.05 mL of Freund’s complete adjuvant (FCA, Sigma, St. Louis, MO, USA) into the sole of the right hind paw in mice; normal saline was injected into the other hind paw (control) [31].

Anti-GcMAF preparation was administered five times at doses ranging from 0.02 ng/mouse to 1.0 μg/mouse on Days 1, 2, (4 or 5), 7, and 9 after arthritis induction. In the experiments using ICR and C57BL/6 mice, anti-GcMAF was administered subcutaneously and intraperitoneally, respectively. Steroidal anti-inflammatory glucocorticoid, dexamethasone (JSC Dalkhimpharm, Khabarovsk, Russia), was chosen as a comparator drug and administered intraperitoneally at a dose of 200 μg/mouse at the same time points as anti-GcMAF. The dose and the dosing schedule of dexamethasone in the preliminary experiment were chosen in accordance with the reported literature data [32]. Control animals received injections of the same saline volume in a similar manner at the same time points as for GcMAF.

The development of inflammation was tested according to difference in thicknesses of experimental and control paws, which were measured using an MCO−25 mm micrometer (JSC “ITO-Tulamash”, Tula, Russia). On the day when the preparation had been administered, it was injected after measuring paw thickness. Follow-up duration in different experiments ranged from 8 to 14 days.

### 2.8. Assessment of the Effect of Anti-GcMAF on Metabolic Activity of Macrophages in Mice

The effect of anti-GcMAF on functional activity of macrophages was assessed using the spectrophotometric nitroblue tetrazolium test [33]. The test was performed according to changes in the level of redox metabolism of cells associated with production of oxygen metabolites (free radicals, hydrogen peroxide, superoxide anions, and singlet oxygen), either spontaneously (I) or via phagocytosis of opsonized ram erythrocytes (II).

### 2.9. Assessment of the Effect of Anti-GcMAF on Hematological Parameters in Mice

White blood cell, red blood cell, and platelet counts as well as hemoglobin and hematocrit levels were measured in blood samples using a MicroCC−20 Plus VET automatic hematology analyzer (HTI Medical Inc., Westminster, MD, USA). The WBC differential was calculated using the standard methods on an EC BIMAM R-11 microscope (JSC “Lomo”, St. Petersburg, Russia) [34].

### 2.10. Induction of Chronic Cystitis in Rats

Cystitis was induced using Endoxan (cyclophosphamide, further referred to as CP), powder for solution for intravenous injection or infusion, 500 mg (Baxter Oncology GmbH, Halle, Germany). For creating the model of chronic cystitis in rats, CP was administered intraperitoneally 3 times with 3-day intervals (on Days 0, 3, and 6) at a dose of 50 mg/kg (10 mg/0.2 mL/200 g of animal body weight).

The method of inducing hemorrhagic cystitis in rats was tested in preliminary experiments involving 27 male rats. The following dosing regimen was selected in accordance with the literature data [35,36,37]: CP was administered three times intraperitoneally on Days 0, 3, and 6 at doses of 25, 40, 75 mg/kg rat body weight; 1, 6, and 10 days after the third injection of CP, rats were euthanized using carbon dioxide and dissected. The bladder was isolated for macroscopic examination, fixed using 10% neutral formalin solution for histological analysis, and transferred for histologic examination.

According to the results of preliminary experiments, the following dosing regimen was chosen for the main experiment to study the effect of anti-GcMAF on the course of chronic cystitis in rats. CP was injected intraperitoneally 3 times every third day (Days 0, 3, and 6) at a dose of 50 mg/1 mL/kg body weight of the animal [37].

### 2.11. Method for Studying the Effect of Anti-GcMAF on the Course of Chronic Cystitis

Anti-GcMAF with the aforementioned characteristics was used in the experiment (Section 3.1) Treatment with anti-GcMAF was started 24 h after the third CP injection (Experimental Day 7). The preparation was injected intraperitoneally five times on Experimental Days 7, 9, 11, 14, and 17. Preparation dose was 1 µg/animal (volume, 200 µL).

Visual examination of the animals was performed daily throughout the entire study period. Rats’ appearance, mobility, behavioral characteristics, food and water consumption, and weight were determined.

On Days 7, 13, 19, and 23 after the first CP injection, the rats were placed into metabolic chambers for urine collection. Urine samples collected during the first 1.5–2 h were divided into two portions. In the first urine portion, we measured such parameters as hemoglobin level, WBC count, acidity (pH), and specific gravity either qualitatively or semi-quantitatively using Uripolian-XN urine test strips. The second portion of urine was centrifuged on a 5810 R centrifuge (Eppendorf SE, Hamburg, Germany) for 15 min at 2500 rpm and 14 °C. The supernatant was collected; 30 μL of the remaining sample was applied onto a glass slide, covered with a coverslip, and the preparation was analyzed using an Axioskop 40 microscope (Carl Zeiss AG, Oberkochen, Germany) under 200× magnification. The quantities of the studied components of urine sediment were recalculated per mL of morning urine sample. In order to determine the daily urine volume, rats were left in the metabolic chamber for 24 h with unlimited access to water.

On Days 7, 13, 20, and 24 after the first administration of CP to animals, blood samples were collected from the retro-orbital sinus to perform hematology and blood chemistry tests. Rats were then euthanized by inhaling carbon dioxide, dissected, and subjected to macroscopic examination. Rat bladders were isolated; their weight was measured on a GX−200 analytical balance (A&D Company, Tokyo, Japan); bladder wall thickness was determined using an MCC 25 digital micrometer (LLC NPP “CHIZ”, Chelyabinsk, Russia). Weight indices of bladders (weight of the organ (mg) divided by animal weight (g) and multiplied by 100) were determined. Each bladder was macroscopically analyzed to assess the condition of blood vessels and mucosal bleeding according to the following criteria using the procedure described in ref. [38]: 0—no vascular damage; 1—minor damage to blood vessels (blood vessels of the mucosa are significantly dilated and filled with blood); 2—moderate damage to blood vessels (sporadic hemorrhages); and 3—severe damage to blood vessels (strip-like hemorrhage on the inner or outer side of the bladder wall; hemorrhagic disease).

Bladders were fixed in 10% neutral formalin solution (BioVitrum, St. Petersburg, Russia) for 48 h to perform histological analysis. The material was treated using the conventional procedure: sequential dehydration in alcohols of increasing concentration, impregnation in xylene–paraffin mixture, and paraffin embedding. Paraffin sections 4–5 µm thick were prepared on an HM−360 automated rotary microtome (Germany). Sections were stained with hematoxylin and eosin. Light optical analysis was carried out on an Axio Imager Z1 microscope (Carl Zeiss AG, Oberkochen, Germany) using the Axio Vision 4.8.2 software (Carl Zeiss AG, Oberkochen, Germany).

WBC, RBC, and platelet counts were determined in blood samples on a MicroCC−20 Plus VET automated hematology analyzer (HTI Medical Inc., Westminster, MD, USA). The WBC differential was calculated using the standard methods on an EC BIMAM R−11 microscope (JSC “Lomo”, St. Petersburg, Russia) [34].

Blood chemistry test involved measuring such parameters as total protein and total cholesterol levels, as well as the levels of albumin urea, glucose, triglycerides, potassium, calcium, chloride, and activities of alanine and aspartate aminotransferase, alkaline phosphatase on a Miura 200 biochemistry analyzer (I.S.E. Ingegneria dei Sistemi Elettronici S.r.l., Vecchiano PI, Italy). Blood chemistry tests were performed in accordance with the instructions of the manufacturer of reagent kits, Vector-Best JSC (Koltsovo, Novosibirsk region, Russia).

### 2.12. Statistical Analysis

The results of experiments to assess cytokine mRNA expression are based on multiple repetitions (at least three replicates). Statistical analysis was performed using the Statistica 10 software (StatSoft, Tulsa, OK, USA). The graphs were designed either using Microsoft Excel 2019 (Microsoft, Redmond, Washington, DC, USA) or GraphPad Prism9.3.1 (Graph-Pad Software, San Diego, CA, USA) software. The graphs show Means ± Standard Deviations (SDs). The validity of differences was evaluated using the Mann–Whitney U test. The revealed differences were considered statistically significant at *p* < 0.05 (Mann–Whitney U test).

## 3. Results

### 3.1. Experimental Search for the Synthesis Mode of GcMAF Exhibiting Anti-Inflammatory Characteristics and Synthesis of Preparative Amounts of Anti-Inflammatory GcMAF

#### 3.1.1. Characterization of mRNA Synthesis by PMs Activated by Exposure to GcMAF Produced by Hydrolysis with Two Enzymes (Sialidase and β–Galactosidase) in the Solution

**Figure 1:**
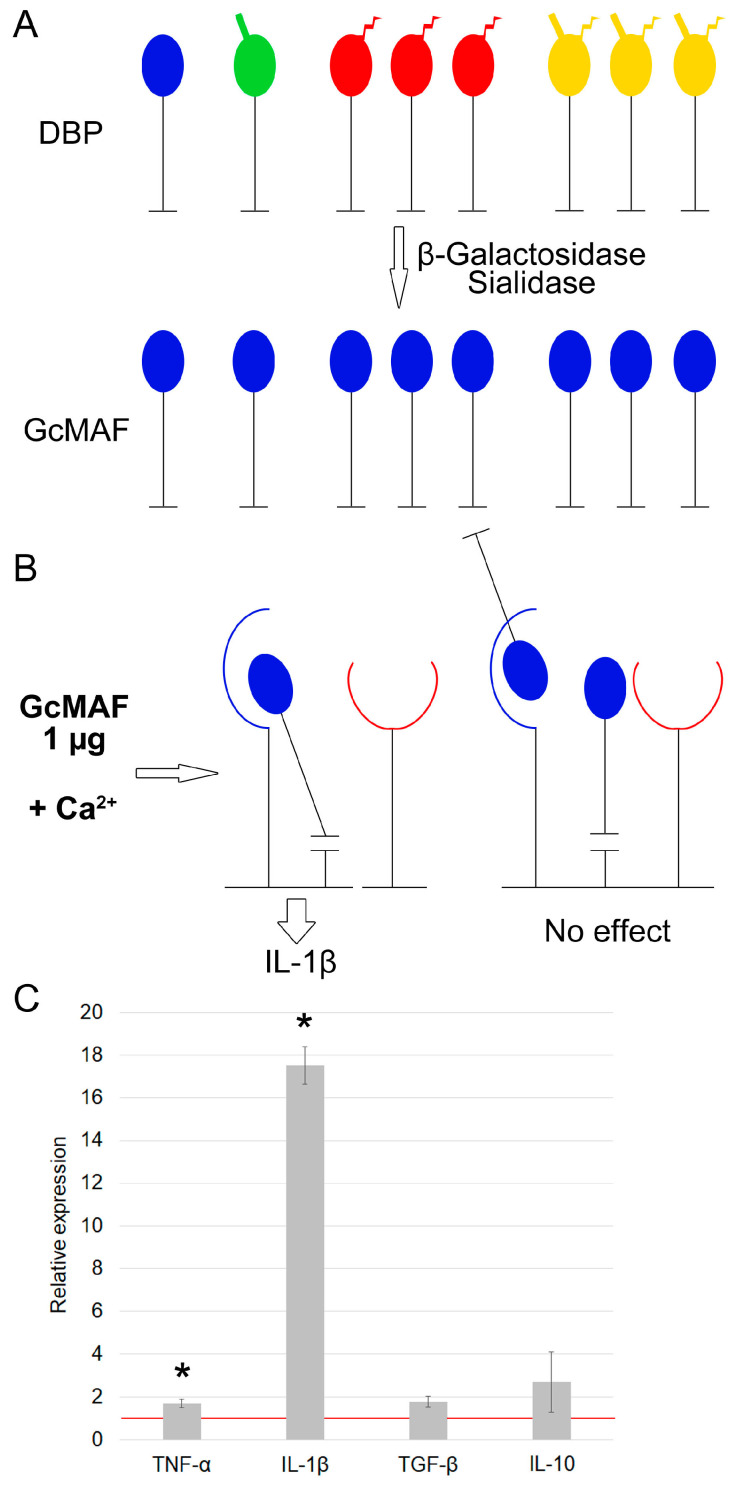
A schematic diagram of the putative mechanism of interaction between pro-GcMAF produced by treatment with two enzymes (β–galactosidase and sialidase) in the solution and CLEC10A, resulting in synthesis of mRNA of analyzed cytokines. A graphical representation of the structural (core) elements used to visualize the interaction between various glycosylated forms of DBP/GcMAF and the CLEC10A complex receptor is provided in Supplementary Figure S1. (**A**) Production of pro-GcMAF carrying a totally open GalNAc moiety by treating all the DBP variants with β–galactosidase and sialidase. (**B**) At a sufficiently high Ca^2+^ concentration, the epitopes of specific Ca^2+^ -dependent 63/65 kDa high-molecular-weight derivatives of the CLEC10A receptor “open up”. When concentration of pro-GcMAF ligand is insufficient, the macrophage-activating factor carrying a terminal GalNAc moiety (shown in blue GalNAc) is bound to both the 29 kDa and 65 kDa derivatives. The complex is internalized into the cytoplasm, and synthesis of proinflammatory cytokines (IL-1β) is activated (the left-hand side of the diagram). Excessive concentration of pro-GcMAF ligand results in competition between the 29 kDa “anchor” derivative and the 65 kDa high-molecular-weight CLEC10A derivative. It is hypothesized that if the doubly bound complex between the ligand and the 29 kDa + 63/65 kDa “anchor” derivatives cannot be formed and internalized into the cytoplasm, cytokine synthesis is inhibited (the right-hand side of the diagram). (**C**) Quantification of mRNA expression of pro- and anti-inflammatory cytokine genes in murine peritoneal macrophages activated by pro-GcMAF at a dose of 1 µg per 5 × 10^5^ PMs. The mRNA expression level in PMs cultured in RPMI−1640 medium assumed to be equal to unity (red solid line) is used as a control. The statistically significant differences compared to the control are denoted as: *—*p* < 0.05, the Mann–Whitney U test.

GcMAF production by hydrolyzing DBP with two enzymes, sialidase and β–galactosidase, results in manifestation of unambiguous synthesis of proinflammatory IL-1β mRNA (Figure 1A–C). Figure 1A shows a visual schematic diagram for the variants of macrophage-activating factor, where all the template molecules after deglycosylation carry only the terminal free GalNAc moiety (shown in blue). Unidirectional binding is possible only for this variant of the composition of deglycosylated molecules, and the findings obtained can be attributed to it (Figure 1B). For a different interpretation, the results cannot be explained (Figure 1C). If there is an excessive amount of protein molecules, some of them are internalized into the cytoplasm within a complex aggregate, while some remain on the surface being engaged with affine modalities (Figure 1B).

#### 3.1.2. Characterization of Properties of Macrophage-Activating Factor in Different Modes of Deglycosylation with β–Galactosidase Monopreparation in the Solution

The following pattern was revealed upon conversion of DBP to GcMAF by β–galactosidase monopreparation, provided that the count of treated macrophages and the volume of the incubation medium were identical in all the experiments.

**Figure 2:**
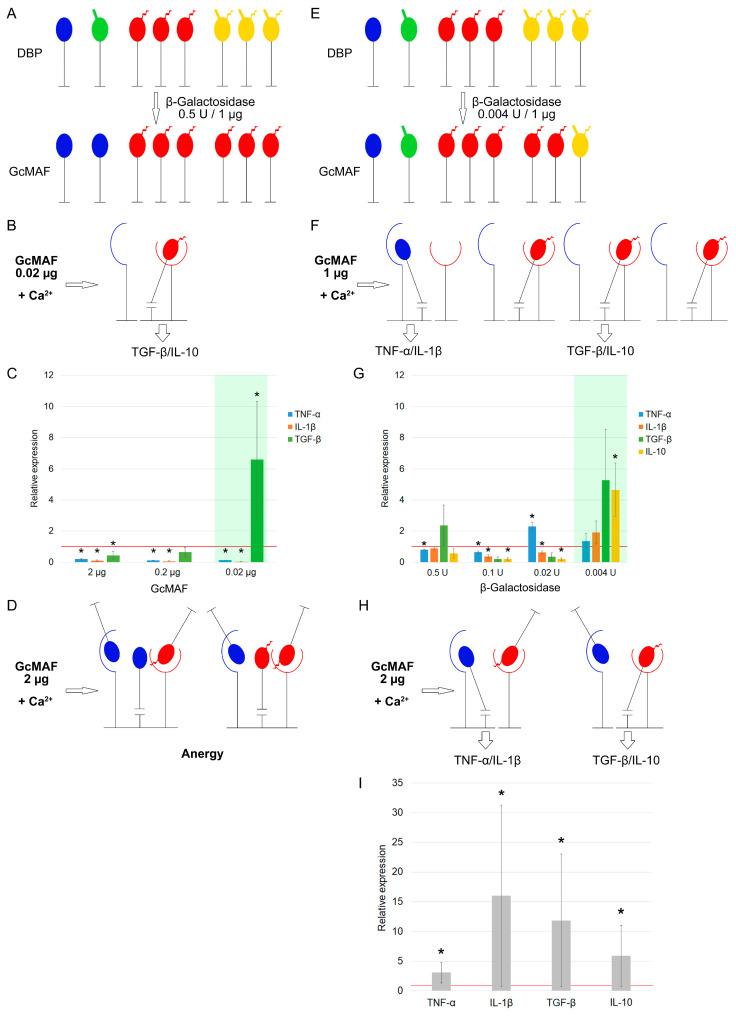
A putative visual schematic diagram showing the effect of co-dependence between the amount of β–galactosidase enzyme used to produce anti-GcMAF from the DBP precursor in solution and the amount of DBP precursor per se on induction of PMs toward mRNA synthesis of the cytokines being analyzed. A graphical representation of the structural (core) elements used to visualize the interaction between various glycosylated forms of DBP/GcMAF and the CLEC10A complex receptor is provided in Supplementary Figure S1. (**A**) A schematic diagram of anti-GcMAF production by converting 1 μg of DBP in the mode “an excessive amount of enzyme per 1 μg of DBP”. (**B**,**C**) For inducing PMs toward synthesis of anti-inflammatory cytokines, the amount of GcMAF produced in the specified mode needs to be 0.02 μg for the cultivation conditions specified in the Materials and Methods section. The green background indicates the mode that produces the anti-inflammatory properties of GcMAF. (**D**) A schematic diagram of inhibition of mRNA synthesis of both pro- and anti-inflammatory cytokines by PMs using an excessive amount of ligand with respective characteristics (2 μg). (**E**) A schematic diagram of anti-GcMAF production by converting 1 μg of DBP in the mode “insufficient amount of enzyme per μg of DBP”. (**F**,**G**) For inducing PMs toward synthesis of anti-inflammatory cytokines, the amount of GcMAF produced in the specified mode needs to be 1.0 μg for the cultivation conditions specified in the Materials and Methods section. The green background indicates the mode that produces the anti-inflammatory properties of GcMAF. (**H**) A schematic diagram of the interaction between the specified amount of GcMAF preparation and CLEC10A on PMs. (**I**) The outcome of mRNA synthesis of analyzed cytokines by PMs for the selected conditions upon excessive amount of the ligand having respective characteristics (2 μg). All the experiments were conducted in the presence of 2 mM Ca^2+^. The results are presented based on the data obtained in three independent experiments. The mRNA expression level in PMs cultured in RPMI−1640 medium assumed to be equal to unity (red solid line) is used as a control. The statistically significant differences compared to the control are denoted as: *—*p* < 0.05, the Mann–Whitney U test.

If an excessive amount of the enzyme is used upon conversion (0.5 U of β–galactosidase per μg of DBP and 0.02 μg of GcMAF produced in the aforementioned conversion mode is taken for activation of PMs), then mRNAs of anti-inflammatory cytokines are synthesized (the count of treated macrophages and incubation medium volumes are identical in all the experiments). Meanwhile, large amounts of GcMAF inhibit synthesis of both pro- and anti-inflammatory cytokines by PMs (Figure 2A–D).

If an insufficient amount of the enzyme is used upon conversion (0.004 U of β–galactosidase per the same amount of protein (1 µg of DBP), while ≥1.0 µg of GcMAF produced in the specified conversion mode is taken for PM activation, mRNA of anti-inflammatory cytokines is also synthesized (the count of treated macrophages and incubation medium volumes are identical in all the experiments) (Figure 2E–G). The excessive amount of GcMAF protein is accompanied by the multidirectional response of PMs toward synthesis of mRNA of pro-/anti-inflammatory cytokines (Figure 2H,I).

Figure 2 shows the mechanistic visual schematic diagram of the process, where the logic of events is as follows. When there is an excessive concentration of the enzyme per µg of DBP, significant amount of protein having the characteristic GalNAc + sialic acid phenotype appears in the sample, thus inducing PMs toward synthesis of anti-inflammatory cytokines. When enzyme concentration per μg of DBP is insufficient, molecules having the GalNAc + sialic acid phenotype appear in the sample, but their amount is insignificant and they are competitively inhibited by an excess of unconverted molecules with non-activating phenotypes. In this case, a large amount of total protein (preparation) subjected to treatment under the aforementioned conditions will be needed for inducing PMs toward synthesis of the same anti-inflammatory cytokines. The small portion of active molecules having the GalNAc + sialic acid phenotype will be compensated for by the fact that their content will be higher in the larger dose of the total preparation. This treatment will also induce mRNA synthesis of anti-inflammatory cytokines by PMs. This very scenario is observed experimentally (Figure 2C,G).

In all the experiments described above, affinity-purified DBP treated with different variants and doses of deglycosylases in a solution was used. β–Galactosidase was found to be a determinant enzyme in manifestation of anti-inflammatory properties of GcMAF produced by precursor conversion.

#### 3.1.3. Characterization of Properties of Macrophage-Activating Factor in Different Modes of Glycosylation with Sialidase Monopreparation in the Solution

**Figure 3:**
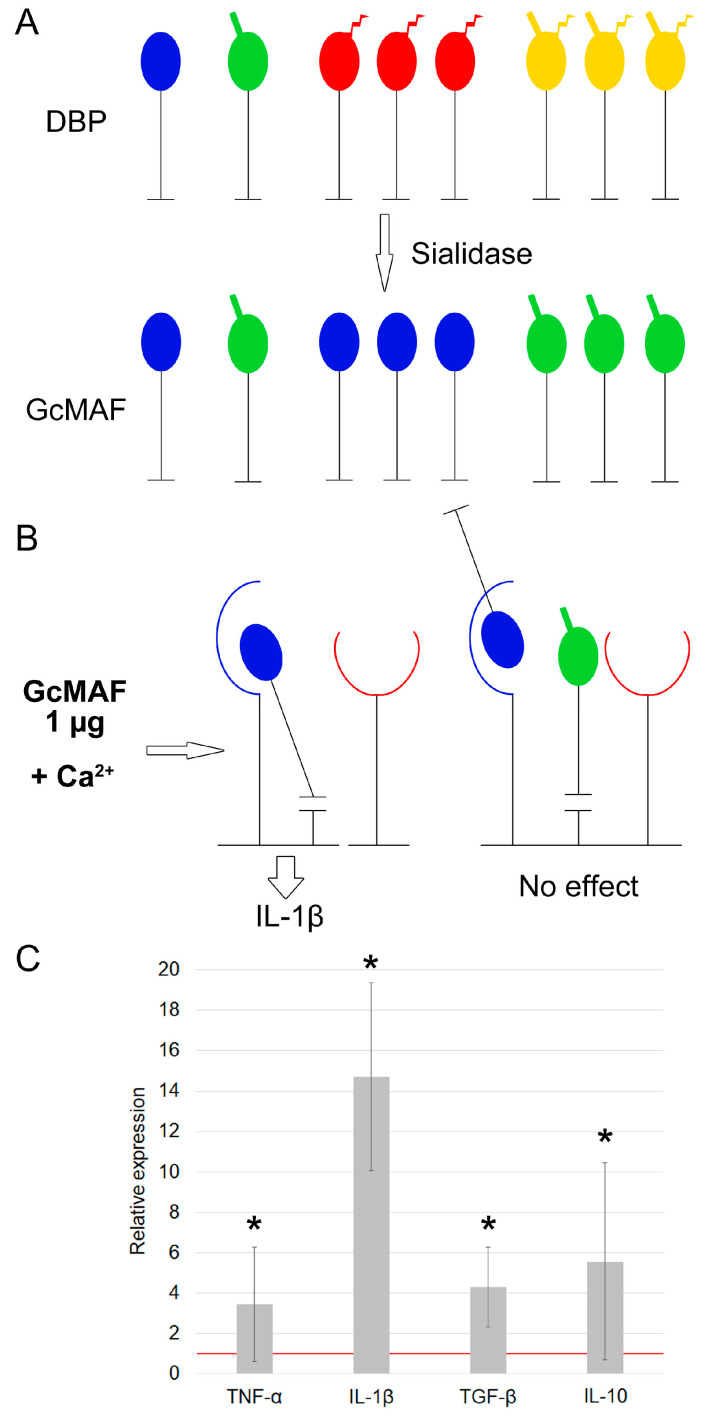
A schematic diagram of the putative mechanism of interaction between pro-GcMAF produced using sialidase in the solution and CLEC10A, resulting in mRNA synthesis of analyzed cytokines by PMs. A graphical representation of the structural (core) elements used to visualize the interaction between various glycosylated forms of DBP/GcMAF and the CLEC10A complex receptor is provided in Supplementary Figure S1. (**A**) Production of pro-GcMAF carrying a GalNAc moiety with a completely opened configuration (shown in blue GalNAc) and carrying GalNAc + galactose (shown in green GalNAc-Gal) by treatment of composite DBP with sialidase at a dose of 0.5 U/μg. (**B**) The epitopes of specific Ca^2+^-dependent 63/65 kDa high-molecular-weight derivatives of the CLEC10A receptor “open up” in the presence of Ca^2+^ ions. At an excessive dose (1 μg), pro-GcMAF carrying a terminal GalNAc moiety (shown in blue) can simultaneously bind to the 29 kDa and 65 kDa derivatives and form a specific complex initiating IL-1b synthesis. Furthermore, partial competition may arise between GcMAF molecules and the GalNAc + galactose deglycosylation variant not activating mRNA synthesis, which are simultaneously present in the mixture under these conditions. In this case, no mRNA synthesis will be observed. Nonetheless, both binding variants will always be present in the mixture and always manifest themselves as mRNA synthesis of IL-1β cytokine by peritoneal macrophages activated with a pro-GcMAF dose of 1 µg. (**C**) Quantification of mRNA expression of pro- and anti-inflammatory cytokine genes in murine peritoneal macrophages activated by pro-GcMAF at a dose of 1 µg per 5 × 10^5^ PMs. The mRNA expression level in PMs cultured in RPMI medium assumed to be equal to unity (red solid line) is used as a control. The statistically significant differences compared to the control are denoted as: *—*p* < 0.05, the Mann–Whitney U test.

Treatment with sialidase yields a large number of molecules in the solution; the molecules carrying free GalNAc induce mRNA synthesis of proinflammatory cytokines, while as mentioned above, those carrying terminal galactose are not involved in induction of PMs toward mRNA synthesis of analyzed cytokines (Figure 3). The pattern of cytokine mRNA synthesis upon activation of PMs using sialidase-treated DBP coincides with that for cytokine mRNA synthesis upon activation of PMs using DBP simultaneously treated with sialidase and β–galactosidase and involves IL-1β mRNA synthesis (Figure 3).

#### 3.1.4. The Effect of Pure Enzymes on mRNA Synthesis of Analyzed Cytokines by PMs

**Figure 4:**
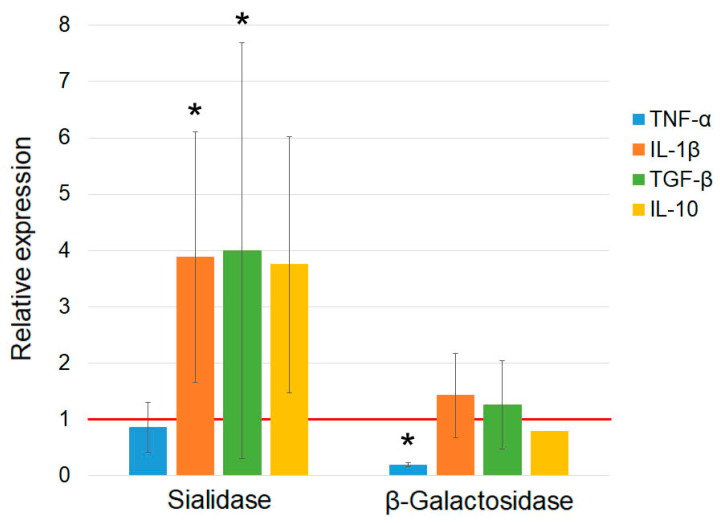
Quantification of mRNA expression of the genes of pro- and anti-inflammatory cytokines by PMs activated with pure sialidase and β–galactosidase enzymes at doses corresponding to 15 mU per 5 × 10^5^ PMs. The mRNA expression level in PMs cultured in RPMI medium assumed to be equal to unity (red solid line) is used as a control. The statistically significant differences compared to the control are denoted as: *—*p* < 0.05, the Mann–Whitney U test.

Assessment of the effect of pure enzymes on mRNA synthesis by PMs indicates that β–galactosidase does not affect cytokine mRNA synthesis, whereas sialidase induces PMs toward synthesis of IL-1β, TGF-β, and IL-10 (Figure 4).

#### 3.1.5. Quantification of β–Galactosidase Dose upon Treatment of DBP “On Resin” That Is Required for Inducing PMs Toward mRNA Synthesis of Anti-Inflammatory Cytokines

As it follows from the experiments, for the anti-inflammatory characteristics of the macrophage-activating factor to be manifested, precursor needs to be converted using β–galactosidase only. Figure 2 summarizes the data obtained by precursor treatment in a solution.

In our previous study [28], the “directly on resin” treatment mode was found to be among the factors for standardizing the procedure for producing the GcMAF preparation.

**Figure 5:**
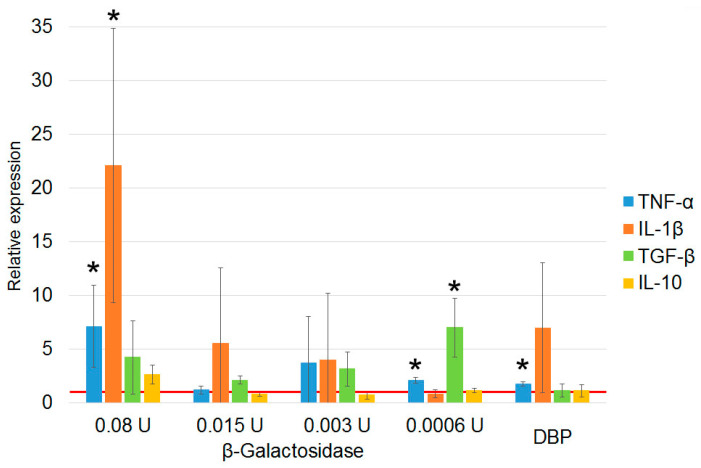
Quantification of mRNA expression of the genes of pro- and anti-inflammatory cytokines by PMs activated using four anti-GcMAF preparations produced by DBP conversion using several β–galactosidase doses: 0.08 U, 0.015 U, 0.003 U, and 0.0006 U per µg of protein “directly on resin” and intact DBP. The mRNA expression level in PMs cultured in RPMI−1640 medium assumed to be equal to unity (red solid line) is used as a control. The statistically significant differences compared to the control are denoted as: *—*p* < 0.05, the Mann–Whitney U test.

Experiments on converting the precursor “directly on resin” using different amounts of β–galactosidase were carried out. The results indicated that only the minimal enzyme dose of 0.0006 U/μg Gc in the “directly on resin” mode induced PMs toward synthesis of anti-inflammatory TGF-β, which corresponded to the mode where conversion was performed in a solution. Other cytokines were not synthesized under these conditions (Figure 5).

### 3.2. The Effect of Anti-Inflammatory GcMAF on Clinical Signs of the Inflammatory Response of Arthrosis in Mice and Hemorrhagic Cystitis in Rats

#### 3.2.1. The Effect of Anti-GcMAF on Clinical Signs of the Inflammatory Response in Mice with Adjuvant Arthritis

**Figure 6:**
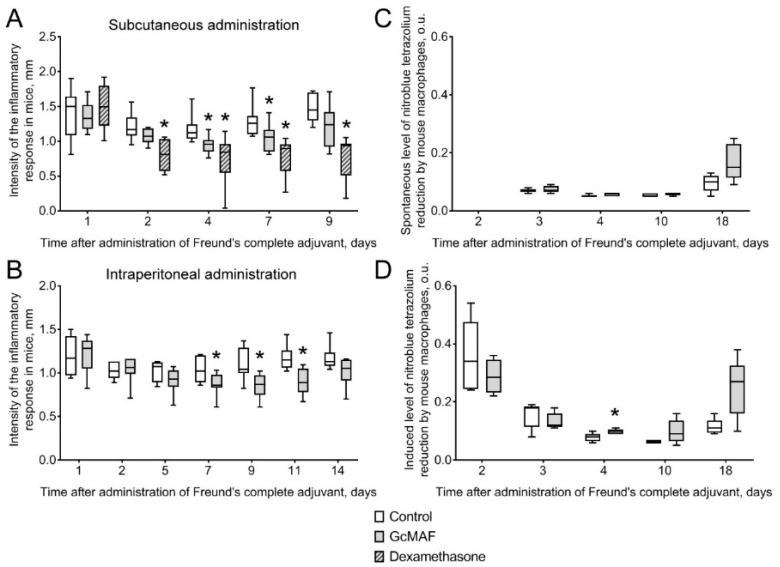
The effect of subcutaneous and intraperitoneal administration of anti-GcMAF at different doses to mice on inflammation intensity and redox activity of peritoneal macrophages induced by administration of FCA. The statistically significant differences compared to the control group are denoted as: *—*p* < 0.05, the Mann–Whitney U test.

A study demonstrated that administration of FCA triggered inflammatory edema in mice (Figure 6A,B). Signs of the inflammatory response were observed 24 h after exposure to FCA and persisted in control animals at approximately the same level for 10 days after the induction of adjuvant arthritis.

Administration of five GcMAF doses reduced inflammation intensity. Statistically significant changes caused by the preparation were detected on Days 2–10 after exposure to FCA. Reduced thickness of the inflamed paw compared to the control parameter during this period was 20–25% (the data are presented in millimeters, Figure 6A,B).

Induction of experimental arthritis was accompanied by increasing redox activity of peritoneal macrophages. The level of NBT (nitro blue tetrazolium) reduction by murine macrophages 24 h after exposure to FCA increased more than fivefold compared to that in intact animals (7.1 ± 0.6 vs. 35.4 ± 5.6) (the data are presented in optical units, Figure 6C,D).

In the acute phase of adjuvant arthritis development, the preparation moderately attenuated the activity of peritoneal macrophages and significantly stimulated the redox metabolism of peritoneal macrophages during the later period of developing symptoms of adjuvant arthritis (Figure 6C,D).

#### 3.2.2. The Effect of Anti-GcMAF on Urine Parameters in Rats with Induced Cystitis

Changes in body weight of rats with hemorrhagic cystitis over time were analyzed. The body weight of rats with chronic cystitis treated with GcMAF did not differ significantly from that of rats in the positive control group (CP, no treatment) during any of the observation periods.

**Figure 7:**
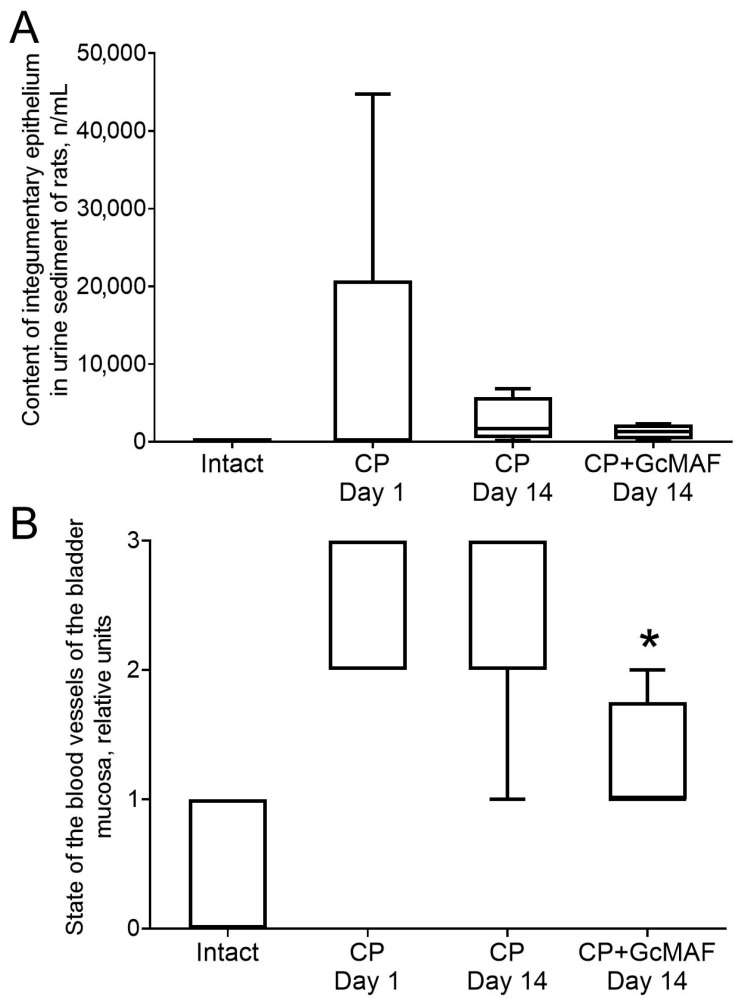
The effect of anti-GcMAF on severity of pathological changes in integumentary epithelium (**A**) and vascular damage of bladder mucosa (**B**) after administration of five doses of anti-GcMAF and cyclophosphamide injection. The statistically significant differences compared to the group (CP Day 14) are denoted as: *—*p* < 0.05, the Mann–Whitney U test.

The results of first morning urine test in rats performed using urine test strips and microscopic examination of urine sediment proved that cystitis was induced in rats after intraperitoneal administration of three doses of CP (each dose being equal to 50 mg/kg body weight). One day after administration of the third dose of CP, rat urine was found to contain white blood cells, red blood cells, integumentary epithelium, protein, and ammonium urate. Figure 7A shows the effect of cyclophosphamide and subsequent administration of GcMAF on the content of integumentary epithelium in rat urine sediment.

#### 3.2.3. Macroscopic and Histological Examination of Bladder Samples Collected from Rats with Induced Hemorrhagic Cystitis

**Figure 8:**
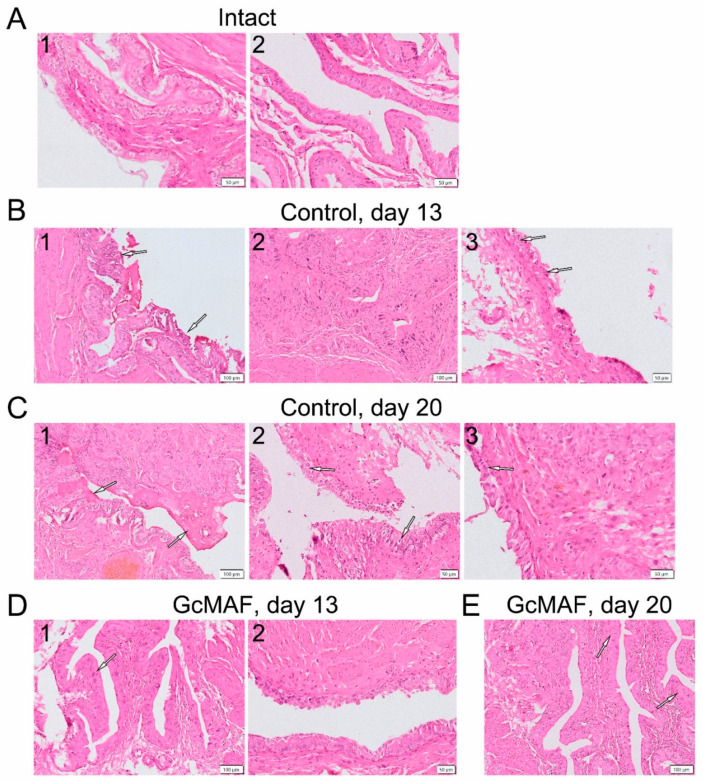
Pathomorphological analysis of the bladder in rats with induced chronic cystitis. (**A**) The normal structure of epithelial lining in intact animals, two independent images. (**B**) Control group on Day 13: (**1**) small areas of epithelial dystrophy and dysplasia, epithelium having normal structure being prevalent; (**2**) marked epithelial dysplasia (nuclear polymorphism); and (**3**) disseminated vacuolar dystrophy of epithelial cells. (**C**) Control group on Day 20. (**1**) blood clots on the urothelial surface (without manifestations of perifocal inflammatory infiltration of cells); (**2**) bright areas (halo) around the epithelial nuclei; and (**3**) the area of epithelial thinning. (**D**) GcMAF, Day 13. (**1**) small epithelial dystrophy and dysplasia areas, epithelium with normal structure being prevalent; (**2**) epithelial vacuolization, extrusion of damaged epitheliocytes, nonuniform epithelial thickness. (**E**) GcMAF, day 20. One can see individual small vacuolar dystrophy regions and epithelial dysplasia-like regions with disrupted differentiation (shown with arrows).

Macroscopic examination of the bladder wall condition showed that administration of three CP doses (50 mg/kg body weight) to rats caused vascular damage of the bladder wall (hemorrhage). Figure 8B summarizes the damage severity assessed according to ref. [38]. The severity of vascular damage in positive control animals (rats that had received CP) remained unchanged starting the first day after induction of hemorrhagic cystitis during two weeks of the observation period. On Day 13 after experiment initiation, after the administration of three anti-GcMAF doses (Day 7 after induction of cystitis) to rats with induced hemorrhagic cystitis, severity of vascular damage did not differ from that for the group of untreated animals with cystitis.

Statistically significant reduction in the severity of vascular damage in the bladder wall by 49% compared to that in the positive control group at this time point and by 55% compared to the group of treated animals on Day 7 of treatment (three injections of GcMAF) was observed after administration of five anti-GcMAF doses (on Day 14 after induction of cystitis and the first injection of GcMAF) (Figure 7B). The resulting findings can demonstrate that anti-GcMAF exhibits a positive effect on the course of hemorrhagic cystitis in rats.

The conducted histological examination of the bladder in rats with induced chronic cystitis demonstrated that the most severe pathological changes after administration of CP in the examined animals were observed on experimental Days 13 and 20 (Days 7 and 14 after cystitis symptoms had been detected) compared to the intact group; these changes involved dysplasia, edema of the proper mucous plate, and urothelial swelling (Figure 8A–C).

Distinct morphological differences in bladder wall condition were observed for animals treated with anti-GcMAF compared to untreated ones (Figure 7A and Figure 8D). On Day 7, after administration of three anti-GcMAF doses, only one out of five animals had significant epithelial structure disruption, whereas in untreated rats with induced cystitis, it was observed in four out of five animals.

After administration of five anti-GcMAF doses (Experimental Day 20), the epithelial structure was nearly normal in three out of four animals (Figure 8E). An exception was one animal from the group that had received treatment: on Experimental Day 20, strongly marked symptoms of acute cystitis accompanied by intense diffuse leukocytic infiltration, probably infectious, of the bladder wall were observed. During this period, marked histopathological changes in integumentary epithelium of the mucosa were detected in all the untreated animals with induced cystitis. The severity of the detected pathological changes was moderate in most animals treated with anti-GcMAF and significant in the group of untreated animals.

#### 3.2.4. Hematological and Biochemical Examination of Blood Collected in Rats with Hemorrhagic Cystitis

During the observation period, hemoglobin level and RBC count in the positive control group and experimental groups were found to decrease compared to those in the intact group. One day after induction of cystitis, we observed leukopenia that was typical of administration of CP; the WBC count subsequently increased after Days 7 and 14. Following the administration of five anti-GcMAF doses (experimental Day 20), the WBC count was statistically significantly higher compared to that in intact animals (by 173%) and did not differ from the positive control (CP). The changes in WBC count were accompanied by a decline in the percentage of neutrophils in WBC differential. The percentage of lymphocytes in both groups statistically significantly decreased 7–14 Days after induction of cystitis. Once treatment had been completed, this parameter was 1.75-fold lower than that for untreated rats with cystitis.

Rats with hemorrhagic cystitis had a statistically significant decline in blood level of total protein in all the observation periods compared to that in the intact group. In treated animals, blood level of total protein after administration of five anti-GcMAF doses returned to the level of intact animals. No statistically significant difference was detected between blood protein level in untreated animals and those treated with five anti-GcMAF doses. Hypoalbuminemia (a decline of 20–40% compared to the intact level) was observed in both experimental groups during all the observation periods.

Administration of CP and anti-GcMAF affected neither blood enzyme activities (alanine aminotransferase, aspartate aminotransferase, and alkaline phosphatase) nor levels of calcium and chlorine ions.

Hence, intraperitoneal administration of three 50 mg/kg doses of CP to rats reduced hemoglobin level and RBC count. It also resulted in a reversible decline in WBC count. Similar changes in parameters over time were observed in rats treated with anti-GcMAF.

The disturbance of blood chemistry parameters in rats with hemorrhagic cystitis manifested itself as a decline in blood protein level because of hypoalbuminemia. In the group of rats treated with anti-GcMAF, total protein level was normalized, while albumin level remained reduced.

## 4. Discussion

### 4.1. Production of GcMAF with Anti-Inflammatory Properties 

Protein glycosylation is one of the fundamental processes responsible for their conformational stability and functional activity. The key functions of proteins related to covalently bound sugar residues is their involvement in activation of immune and stem cells, intercellular adhesion, interaction between viral glycoproteins and target cells. Both cell surface receptors and their ligands can be glycosylated. The directions of cellular response to interaction between glycosylated cell surface proteins and their ligands vary strongly: synthesis of cytokine array by immune cells is induced, gene cascades regulating differentiation of precursor stem cells are initiated, lytic properties of killer cells are activated. Glycosylation contributes to immune evasion by tumor cells and their survival during anoikis and determines cell adhesion and related intertissue migration processes [39,40,41,42].

Interaction between glycosylated GcMAF and specific CLEC10A (MGL) is one of the examples of interaction between proteins carrying the glycosylation site with a receptor involved in activation of specific properties of a certain cell type (macrophages).

The mechanisms of inducing professional properties of macrophages are diverse and primarily involve the receptor-mediated induction of immune and pro-/anti-inflammatory responses of phagocytes, which consists in production of specific cytokines, chemokines and factors by them [43]. The direction of inflammatory responses depends on the macrophage phenotype: M2—anti- and M1—pro-inflammatory macrophages. Furthermore, there also is the mixed M1/M2 phenotype, the M0 phenotype (inactivated cells), as well as significant functional diversity of phagocyte subtypes [25].

As mentioned in the Introduction section, the precursor of macrophage-activating factor, DBP, carries a trisaccharide whose deglycosylation degree determines the direction of inflammatory activation of murine PMs. In the present study, we used the macrophage-activating factor GcMAF produced by enzymatic hydrolysis of the DBP precursor with β–galacosidase and sialidase to assess mRNA synthesis of pro-inflammatory mediators, IL-1β and TNF-α, and the two major anti-inflammatory cytokines, TGF-β and IL-10, by murine PMs. It was shown that mRNA synthesis of proinflammatory IL-1β, and in some cases TNF-α, is activated for almost any combination of deglycosylases, and only in some variants, mRNAs of anti-inflammatory cytokines analyzed are synthesized either simultaneously with these mRNAs or individually.

A lectin-type receptor (CLEC10A or MGL) is present on macrophages and dendritic cells and is a regulatory molecule [42]. The interaction between this receptor and a specific ligand (trisaccharide and its terminal hydrolytic derivatives) induces various intracellular responses and processes. It is quite possible that the biological features of C-type lectin receptor–GcMAF engagement modulate activity of PMs. Thus, receptor–ligand engagement is known to alter expression of the genes encoding the key enzymes involved in glycolysis, tricarboxylic acid cycle, and oxidative phosphorylation of phagocytes [44]. For different macrophage subpopulations, metabolic activity is regulated in a differentiated manner. Activity of glycolytic enzymes is high for proinflammatory M1 cells, whereas the tricarboxylic acid cycle is active in the case of M2 macrophages [45,46]. The interaction betweenCLEC10A and Tn antigen on a CD45 molecule of effector T cells causes their anergy and apoptosis [47].

The CLEC10A receptor binds three calcium molecules and the ligand to form clusters and is internalized into the intracellular compartments [42,48]. The ligand-activated C-type lectin receptor was shown to stimulate IL-10 production and simultaneously, through cobinding to TLRs, induce TLR-mediated TNF-α production [49,50]. This fact suggests the dual role of macrophages in inflammatory processes.

The multidirected inflammatory response of cells was also revealed in our study through analysis of the cytokine-stimulating effect of GcMAF with different deglycosylation degrees. GcMAF is believed to carry a trisaccharide at 418Thr or 420Thr positions of the core protein molecule, which consists of GalNAc linked to Thr by an O-glycosidic bond and galactose and sialic acid residues that are linked to the carbohydrate in an either branched or linear form. Depending on enzymatic treatment, murine peritoneal macrophages were found to acquire either a pro- or an anti-inflammatory phenotype, thus synthesizing mRNA of proinflammatory IL-1 β, TNF-α or anti-inflammatory IL-10, TGF-β cytokines, respectively; mRNA of these cytokines can also be synthesized simultaneously (that is, the cells have a mixed M1/M2 phenotype). In our model, β–galactosidase is apparently the key enzyme responsible for the direction of inflammatory response of phagocytes. This effect depends on the quantitative ratio between the target GcMAF and the enzyme and is associated with the emergence of free GalNAc moiety or GalNAc–sialic acid disaccharide. This fact agrees with the data demonstrating that the C-type lectin receptor can interact with both sialylated and nonsialylated antigen [51,52]. The feasibility of coactivation of both CLEC10A and TLR by the same GcMAF molecule on the same cell hinders production of GcMAF having a unidirectional direction of anti-inflammatory activity. As demonstrated in our previous study, the direction of inflammatory response seems to depend on the variant of engagement between saccharide residue and one of the high-molecular-weight derivatives of CLEC10A (65 kDa or 63 kDa one) (for details see Section 2.1). According to our findings, binding to the canonical 29 kDa CLEC10A is independent of the presence of Ca^2+^ ions and is determined by interaction between the backbones of ligand and receptor molecules. A similar interaction variant was shown in ref. [53], where along with the GalNAc binding domain, a second interaction site residing in the body of a protein molecule was shown for CLEC10A. Furthermore, binding of α- or β– isomers of GalNAc [54] to CLEC10A induces the fundamental and unique changes in the conformation of the CLEC10A molecule depending on the GalNAc structure [52]. It implies that ligand-mediated internalization of CLEC10A may induce activation of different signaling pathways (ERK/MAPK, NF-κB, PI3K-Akt, and/or PLCγ2), thus affecting biological processes in a different way depending on the ligand structure [42,48,52,55,56].

By analyzing different uncontrolled forms of macrophage activation by GcMAF that were revealed in our studies, it is fair to assume that variation of conformations of the sugar residue in GcMAF is one of the ways for modulating the direction of inflammatory responses of PMs.

As mentioned above, the receptor can form clusters (that is, be aggregated after being activated by the ligand) [48]. The present study has repeatedly demonstrated that synthesis of any mRNA is inhibited in the presence of an excess of GcMAF, which is probably associated with receptor aggregation as evidenced by cytological data obtained by us [28].

The following conclusions can be drawn to summarize the results of this study. GcMAF obtained by simultaneous treatment of DBP with two enzymes in a solution induces murine PMs toward synthesis of exclusively proinflammatory IL-1β. Treatment with sialidase also induces murine PMs toward synthesis of proinflammatory IL-1β and, to a minor extent, IL-10. In the monomode, the enzyme insignificantly activates mRNA synthesis of IL-1β, TGF-β, and IL-10 by PMs.

Treatment with β–galactosidase leads to a complex pattern of response from PMs.

When an excessive amount of the enzyme with respect to the precursor was used, a specific rise in TGF-β mRNA expression was observed in one of the titrated doses. A similar result and a rise in IL-10 mRNA expression was observed when the protein was exposed to an insufficient amount of the enzyme. In both cases, there appears a free sialic acid residue due to which synthesis of mRNA of anti-inflammatory cytokines is presumably induced. Furthermore, if the solution contains two protein forms with terminal GalNAc and GalNAc–sialic acid moieties, two types of phagocytic responses are possible. Competitive binding to a tripartite receptor may either lead to receptor aggregation and clusterization or induce synthesis of both pro- and anti-inflammatory cytokines as revealed by our numerous experimental studies.

It follows from the conducted analysis that because of the uncontrolled multifactorial conditions affecting the outcome of the CLEC10A receptor–GcMAF ligand interaction, targeted exhaustive search with conditions approximating those yielding a predicted form of response from PMs, is an acceptable approach to acquiring the desired form of the inflammatory response of the phagocyte system.

Brief conclusions:

1. Hence, mRNA expression of pro- or anti-inflammatory cytokine genes is affected by the composition of the population of GcMAF molecules and the content of different variants of glycosylation sites in this population interacting with CLEC10 derivatives.
2. In the presence of an excessive amount of GcMAF molecules, their aggregation (or other processes) occurs, and mRNA synthesis of the analyzed cytokines stops.
3. The galactose terminal residue does not affect mRNA synthesis. Molecules carrying a terminal galactose residue compete for landing on the “anchor” 29 kDa CLEC10 derivative.
4. The GalNAc moiety carrying a terminal sialic acid residue binds to the 63 kDa CLEC10 and induces TGF-β/IL-10 synthesis [28].
5. The terminal GalNAc moiety interacts with 65 kDa CLEC10 and induces IL-1β synthesis [28].

It is different quantitative combinations of these molecules that control synthesis direction.

### 4.2. The Effect of GcMAF with Anti-Inflammatory Properties on Models of Induced Arthritis in Mice and Cystitis in Rats

Elaboration of conditions for producing preparative amounts of GcMAF exhibiting anti-inflammatory characteristics enabled in vivo experiments for assessing the effect of injecting this preparation on inflammation induction. Two experimental models were chosen: induced acute arthritis in mice and induced acute cystitis in rats (see the Materials and Methods section). Preparation inducing PMs toward IL-10 synthesis was selected for the experiments.

In vivo experiments conducted to assess the effect of anti-inflammatory GcMAF on clinical signs of the inflammatory response in models of induced acute arthritis and cystitis indicate that exposure to the preparation elicits a statistically significant anti-inflammatory effect. Experiments in mice with adjuvant arthritis experimentally induced by exposure to Freund’s complete adjuvant revealed that anti-GcMAF injected intraperitoneally and subcutaneously to mice at a dose of 1 µg/mouse inhibited local inflammatory response. Reduction of inflammation upon exposure to anti-GcMAF at lower doses (0.1 and 0.01 μg/mouse) was observed after a longer period. Administration of anti-GcMAF to mice with adjuvant arthritis alters the functional state of immune cells involved in the inflammatory response. The effect of preparation on the function of murine peritoneal macrophages was multidirectional, being potentially related to the inflammation development phase. During the acute inflammation period, anti-GcMAF moderately reduced the redox metabolism indices of macrophages; during the later period of arthritis development, GcMAF stimulated the metabolic activity of macrophages.

GcMAF (five doses) administered intraperitoneally to rats with hemorrhagic cystitis induced by CP was shown to exhibit a therapeutic effect manifesting itself in restoration of bladder wall epithelium and normalization of the vascular state.

The results obtained in this section and the findings reported in our recent publications [28,29] indicate that anti-inflammatory GcMAF can contribute to reduction of inflammation of different etiologies at the organism level due to induction of macrophage-mediated immunity (different types of macrophages: peritoneal macrophages, alveolar macrophages, Kupffer cells, and microglia) toward synthesis of anti-inflammatory cytokines TGF-β and IL-10.

It is known that in the case of rheumatoid arthritis, interactions occur between a variety of cells (in particular, macrophages, T and B cells, synovial fibroblasts, as well as mast, dendritic and plasma cells) in the inflamed synovial membrane of joints, causing a release of proinflammatory mediators (TNF-α, IL-1β, and IL-6) [57]. Interstitial cystitis causes damage to bladder epithelium, increased barrier permeability, neurogenic inflammation with mast cell infiltration, and potential autoimmune involvement [58,59]. Since GcMAF activates the immune response associated with macrophage activity, it can affect the pathogenesis of rheumatoid arthritis and interstitial cystitis through specific activity of macrophages. GcMAF is a potential medicine for pathogenetic therapy of inflammation of different etiologies.

## 5. Conclusions

We believe that findings obtained in this study and being indicative of selective macrophage response to activation by specific GcMAF whose properties are dependent on the structure of its glycosylation site should be taken into account in further research.

The revealed algorithm of producing GcMAF exhibiting anti-inflammatory properties suggests that this MAF can be used in a targeted manner for treating diseases related to inflammation induced by macrophage-mediated immunity of different etiology.

The key research problem requiring further experiments is the question related to the molecular mechanisms of such differentiated macrophage response to activation by GcMAF carrying specific carbohydrate composition. It is also an open question whether similar changes in the glycosylation site structure can modify the direction of immune response of other cell types whose activation depends on interaction between lectin receptor and a carbohydrate-bearing ligand.

## 6. Patents

The method used for producing the GcMAF preparation has been patented (priority No. 047390 2023121663 dated 17 August 2023).

## Data Availability

The data supporting the findings of this study are available from the corresponding author upon reasonable request.

## Supplementary Materials

The following supporting information can be downloaded at: https://www.mdpi.com/article/10.3390/cimb46100650/s1.

## References

[1] Yang F., Brune J.L., Naylor S.L., Cupples R.L., Naberhaus K.H., Bowman B.H. (1985). Human Group-Specific Component (Gc) Is a Member of the Albumin Family. Proc. Natl. Acad. Sci. USA.

[2] Morales E. (2017). GcMAF: A Polemic or a Highly Promising Molecule?. World Sci. News.

[3] Saburi E., Saburi A., Ghanei M. (2017). Promising Role for Gc-MAF in Cancer Immunotherapy: From Bench to Bedside. Casp. J. Intern. Med..

[4] Albracht S.P. (2022). Immunotherapy with GcMAF Revisited—A Critical Overview of the Research of Nobuto Yamamoto. Cancer Treat. Res. Commun..

[5] Ruggiero M., Ward E., Smith R., Branca J.J.V., Noakes D., Morucci G., Taubmann M., Thyer L., Pacini S. (2014). Oleic Acid, Deglycosylated Vitamin D-Binding Protein, Nitric Oxide: A Molecular Triad Made Lethal to Cancer. Anticancer Res..

[6] Uto Y., Yamamoto S., Takeuchi R., Nakagawa Y., Hirota K., Terada H., Onizuka S., Nakata E., Hori H. (2011). Effect of the Gc-Derived Macrophage-Activating Factor Precursor (PreGcMAF) on Phagocytic Activation of Mouse Peritoneal Macrophages. Anticancer Res..

[7] Yamamoto N., Homma S. (1991). Vitamin D3 Binding Protein (Group-Specific Component) Is a Precursor for the Macrophage-Activating Signal Factor from Lysophosphatidylcholine-Treated Lymphocytes. Proc. Natl. Acad. Sci. USA.

[8] Nagasawa H., Uto Y., Sasaki H., Okamura N., Murakami A., Kubo S., Kirk K.L., Hori H. (2005). Gc Protein (Vitamin D-Binding Protein): Gc Genotyping and GcMAF Precursor Activity. Anticancer Res..

[9] Viau M., Constans J., Debray H., Montreuil J. (1983). Isolation and Characterization of the O-Glycan Chain of the Human Vitamin-D Binding Protein. Biochem. Biophys. Res. Commun..

[10] Yamamoto N., Kumashiro R. (1993). Conversion of Vitamin D3 Binding Protein (Group-Specific Component) to a Macrophage Activating Factor by the Stepwise Action of Beta-Galactosidase of B Cells and Sialidase of T Cells. J. Immunol..

[11] Homma S., Yamamoto M., Yamamoto N. (1993). Vitamin D-Binding Protein (Group-Specific Component) Is the Sole Serum Protein Required for Macrophage Activation after Treatment of Peritoneal Cells with Lysophosphatidylcholine. Immunol. Cell Biol..

[12] Naraparaju V.R., Yamamoto N. (1994). Roles of β-Galactosidase of B Lymphocytes and Sialidase of T Lymphocytes in Inflammation-Primed Activation of Macrophages. Immunol. Lett..

[13] Yamamoto N., Homma S., Haddad J.G., Kowalski M.A. (1991). Vitamin D3 Binding Protein Required for in Vitro Activation of Macrophages after Alkylglycerol Treatment of Mouse Peritoneal Cells. Immunology.

[14] Cooke N.E., David E.V. (1985). Serum Vitamin D-Binding Protein Is a Third Member of the Albumin and Alpha Fetoprotein Gene Family. J. Clin. Investig..

[15] Cleve H., Constans J. (1988). The Mutants of the Vitamin-D-Binding Protein: More than 120 Variants of the GC/DBP System. Vox Sang..

[16] Morita Y., Wang R., Li X., Muramatsu T., Ueda M., Hachimura S., Takahashi S., Miyakawa T., Tanokura M. (2020). Improved Preparation of Group-Specific Component (Gc) Protein to Derive Macrophage Activating Factor. Protein Expr. Purif..

[17] Uto Y., Hori H., Kubo K., Ichihashi M., Sakamoto N., Mette M., Inui T. (2012). GcMAF: Our next-Generation Immunotherapy. Nature.

[18] Borges C.R., Jarvis J.W., Oran P.E., Nelson R.W. (2008). Population Studies of Vitamin D Binding Protein Microheterogeneity by Mass Spectrometry Lead to Characterization of Its Genotype-Dependent O-Glycosylation Patterns. J. Proteome Res..

[19] Ravnsborg T., Olsen D.T., Thysen A.H., Christiansen M., Houen G., Højrup P. (2010). The Glycosylation and Characterization of the Candidate Gc Macrophage Activating Factor. Biochim. Biophys. Acta-Proteins Proteom..

[20] Malik S., Fu L., Juras D.J., Karmali M., Wong B.Y.L., Gozdzik A., Cole D.E.C. (2013). Common Variants of the Vitamin D Binding Protein Gene and Adverse Health Outcomes. Crit. Rev. Clin. Lab. Sci..

[21] Daiger S.P., Schanfield M.S., Cavalli Sforza L.L. (1975). Group-Specific Component (Gc) Proteins Bind Vitamin D and 25-Hydroxyvitamin, D. Proc. Natl. Acad. Sci. USA.

[22] Borges C.R., Rehder D.S. (2016). Glycan Structure of Gc Protein-Derived Macrophage Activating Factor as Revealed by Mass Spectrometry. Arch. Biochem. Biophys..

[23] Rehder D.S., Nelson R.W., Borges C.R. (2009). Glycosylation Status of Vitamin D Binding Protein in Cancer Patients. Protein Sci..

[24] Kanie Y., Maegawa Y., Wei Y., Kanie O. (2023). Investigation of the Protective Effect for GcMAF by a Glycosidase Inhibitor and the Glycan Structure of Gc Protein. Molecules.

[25] Lee K.Y. (2019). M1 and M2 Polarization of Macrophages: A Mini-Review. Med. Biol. Sci. Eng..

[26] Ruggiero M. (2016). Gc Protein-Derived Macrophage Activating Factor (GcMAF) and Autism: Do Clinical Results Require a Novel Interpretation?. Am. J. Immunol..

[27] Dolgova E.V., Kirikovich S.S., Levites E.V., Ruzanova V.S., Proskurina A.S., Ritter G.S., Taranov O.S., Varaksin N.A., Ryabicheva T.G., Leplina O.Y. (2022). Analysis of the Biological Properties of Blood Plasma Protein with GcMAF Functional Activity. Int. J. Mol. Sci..

[28] Kirikovich S.S., Levites E.V., Proskurina A.S., Ritter G.S., Peltek S.E., Vasilieva A.R., Ruzanova V.S., Dolgova E.V., Oshihmina S.G., Sysoev A.V. (2023). The Molecular Aspects of Functional Activity of Macrophage-Activating Factor GcMAF. Int. J. Mol. Sci..

[29] Ruzanova V.S., Kirikovich S.S., Levites E.V., Proskurina A.S., Dolgova E.V., Ritter G.S., Efremov Y.R., Dubatolova T.D., Sysoev A.V., Koleno D.I. (2024). The Macrophage Activator GcMAF-RF Enhances the Antitumor Effect of Karanahan Technology through Induction of M2-M1 Macrophage Reprogramming. J. Immunol. Res..

[30] Bouillon R., Schuit F., Antonio L., Rastinejad F. (2020). Vitamin D Binding Protein: A Historic Overview. Front. Endocrinol..

[31] Chillingworth N.L., Donaldson L.F. (2003). Characterisation of a Freund’s Complete Adjuvant-Induced Model of Chronic Arthritis in Mice. J. Neurosci. Methods.

[32] Bogacheva N.V., Tuneva N.A., Smirnov A.A., Galyamova D.A., Popescu L.I. (2018). Development of a biological model of immunosuppression using dexamethasone. Vyatka Med. Bull..

[33] Madzhidov U.V. (1984). Suppressor T Cells upon Experimentally Induced Adjuvant Arthritis and Immune Suppression. Zh. Mikrobiol. Epidemiol. Immunobiol..

[34] Novitskii V.V., Evtushenko O.M. (1999). Practical Hematology Guidelines.

[35] Chen J.L., Zhou X., Ding H.L., Zhan H.L., Yang F., Li W.B., Xie J.C., Liu X.G., Xu Y.C., Su M.Z. (2019). Neuregulin-1-ErbB Signaling Promotes Microglia Activation Contributing to Mechanical Allodynia of Cyclophosphamide-Induced Cystitis. Neurourol. Urodyn..

[36] Ding H., Chen J., Su M., Lin Z., Zhan H., Yang F., Li W., Xie J., Huang Y., Liu X. (2020). BDNF Promotes Activation of Astrocytes and Microglia Contributing to Neuroinflammation and Mechanical Allodynia in Cyclophosphamide-Induced Cystitis. J. Neuroinflammation.

[37] Augé C., Gamé X., Vergnolle N., Lluel P., Chabot S. (2020). Characterization and Validation of a Chronic Model of Cyclophosphamide-Induced Interstitial Cystitis/Bladder Pain Syndrome in Rats. Front. Pharmacol..

[38] Gray K.J., Engelmann U.H., Johnson E.H., Fishman I.J. (1986). Evaluation of Misoprostol Cytoprotection of the Bladder with Cyclophosphamide (Cytoxan) Therapy. J. Urol..

[39] Varki A., Cummings R.D., Esko J.D., Freeze H.H., Stanley P., Bertozzi C.R., Hart G.W., Etzler M.E. (2009). Essentials of Glycobiology. Cold Spring Harb..

[40] Lima M., Baynes J.W. (2013). Glycation. Encyclopedia of Biological Chemistry.

[41] Takeuchi H., Haltiwanger R.S. (2014). Significance of Glycosylation in Notch Signaling. Biochem. Biophys. Res. Commun..

[42] Hoober K.J. (2020). Asgr1 and Its Enigmatic Relative, CLEC10A. Int. J. Mol. Sci..

[43] Klimp A.H., De Vries E.G.E., Scherphof G.L., Daemen T. (2002). A Potential Role of Macrophage Activation in the Treatment of Cancer. Crit. Rev. Oncol. Hematol..

[44] Zaal A., Li R.J.E., Lübbers J., Bruijns S.C.M., Kalay H., van Kooyk Y., van Vliet S.J. (2020). Activation of the C-Type Lectin MGL by Terminal GalNAc Ligands Reduces the Glycolytic Activity of Human Dendritic Cells. Front. Immunol..

[45] Jha A.K., Huang S.C.C., Sergushichev A., Lampropoulou V., Ivanova Y., Loginicheva E., Chmielewski K., Stewart K.M., Ashall J., Everts B. (2015). Network Integration of Parallel Metabolic and Transcriptional Data Reveals Metabolic Modules That Regulate Macrophage Polarization. Immunity.

[46] Geeraerts X., Bolli E., Fendt S.M., Van Ginderachter J.A. (2017). Macrophage Metabolism as Therapeutic Target for Cancer, Atherosclerosis, and Obesity. Front. Immunol..

[47] van Vliet S.J., Gringhuis S.I., Geijtenbeek T.B.H., van Kooyk Y. (2006). Regulation of Effector T Cells by Antigen-Presenting Cells via Interaction of the C-Type Lectin MGL with CD45. Nat. Immunol..

[48] Napoletano C., Zizzari I.G., Rughetti A., Rahimi H., Irimura T., Clausen H., Wandall H.H., Belleudi F., Bellati F., Pierelli L. (2012). Targeting of Macrophage Galactose-Type C-Type Lectin (MGL) Induces DC Signaling and Activation. Eur. J. Immunol..

[49] van Vliet S.J., Bay S., Vuist I.M., Kalay H., García-Vallejo J.J., Leclerc C., van Kooyk Y. (2013). MGL Signaling Augments TLR2-Mediated Responses for Enhanced IL-10 and TNF-α Secretion. J. Leukoc. Biol..

[50] Heger L., Balk S., Lühr J.J., Heidkamp G.F., Lehmann C.H.K., Hatscher L., Purbojo A., Hartmann A., Garcia-Martin F., Nishimura S.I. (2018). CLEC10A Is a Specific Marker for Human CD1c+ Dendritic Cells and Enhances Their Toll-like Receptor 7/8-Induced Cytokine Secretion. Front. Immunol..

[51] Mortezai N., Behnken H.N., Kurze A.K., Ludewig P., Buck F., Meyer B., Wagener C. (2013). Tumor-Associated Neu5Ac-Tn and Neu5Gc-Tn Antigens Bind to C-Type Lectin CLEC10A (CD301, MGL). Glycobiology.

[52] Diniz A., Coelho H., Dias J.S., van Vliet S.J., Jiménez-Barbero J., Corzana F., Cabrita E.J., Marcelo F. (2019). The Plasticity of the Carbohydrate Recognition Domain Dictates the Exquisite Mechanism of Binding of Human Macrophage Galactose-Type Lectin. Chem.-A Eur. J..

[53] Marcelo F., Supekar N., Corzana F., Van Der Horst J.C., Vuist I.M., Live D., Boons G.J.P.H., Smith D.F., Van Vliet S.J. (2019). Identification of a Secondary Binding Site in Human Macrophage Galactose-Type Lectin by Microarray Studies: Implications for the Molecular Recognition of Its Ligands. J. Biol. Chem..

[54] Wang J., Zhang W., Cao W., Liu K., Su S., Ma J., Li X. (2022). Selective Synthesis of α- and β-Glycosides of N-Acetyl Galactosamine Using Rare Earth Metal Triflates. Front. Chem..

[55] Munkley J., Elliott D.J., Munkley J., Elliott D.J. (2016). Hallmarks of Glycosylation in Cancer. Oncotarget.

[56] Gu C., Wang L., Zurawski S., Oh S. (2019). Signaling Cascade through DC-ASGPR Induces Transcriptionally Active CREB for IL-10 Induction and Immune Regulation. J. Immunol..

[57] Udalova I.A., Mantovani A., Feldmann M. (2016). Macrophage Heterogeneity in the Context of Rheumatoid Arthritis. Nat. Publ. Gr..

[58] Homma Y., Ueda T., Tomoe H., Lin A.T.L., Kuo H.C., Lee M.H., Oh S.J., Kim J.C., Lee K.S. (2016). Clinical Guidelines for Interstitial Cystitis and Hypersensitive Bladder Updated in 2015. Int. J. Urol..

[59] Birder L.A. (2019). Pathophysiology of Interstitial Cystitis. Int. J. Urol..

