# Supplementary material for: Production of GcMAF with Anti-Inflammatory Properties and Its Effect on Models of Induced Arthritis in Mice and Cystitis in Rats
Source: Curr Issues Mol Biol. 2024 Sep 28;46(10):10934–59. doi: 10.3390/cimb46100650 (PMC11506609; doi:10.3390/cimb46100650)
Supplement: Supplementary file 1 [file cimb-46-00650-s001.zip › cimb-3204242-supplementary.pdf]

Supplementary Figures

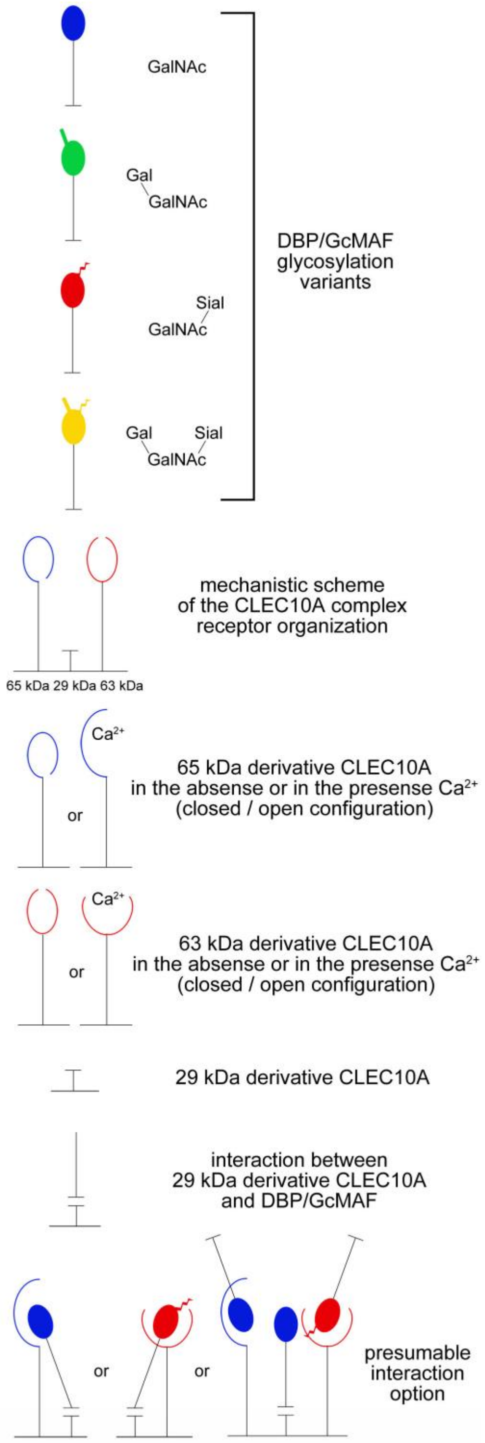

Figure S1: Graphical representation of the structural elements used to visualize the interaction variants between different glycosylated forms of DBP/GcMAF and the CLEC10A complex receptor. Variants of glycosylated DBP/GcMAF forms present in plasma with allowance for the available literature data (see explanation in the text). It is supposed that some DBP/GcMAF molecules carry GalNAc only (shown in blue GalNAc); some of them carry GalNAc + galactose (shown in green GalNAc-Gal); some of them carry GalNAc + sialic acid (shown in red GalNAc-Sial); or glycosylated molecules carrying all three sugar residues (GalNAc + galactose + sialic acid) are present (shown in yellow GalNAc-Gal-Sial).

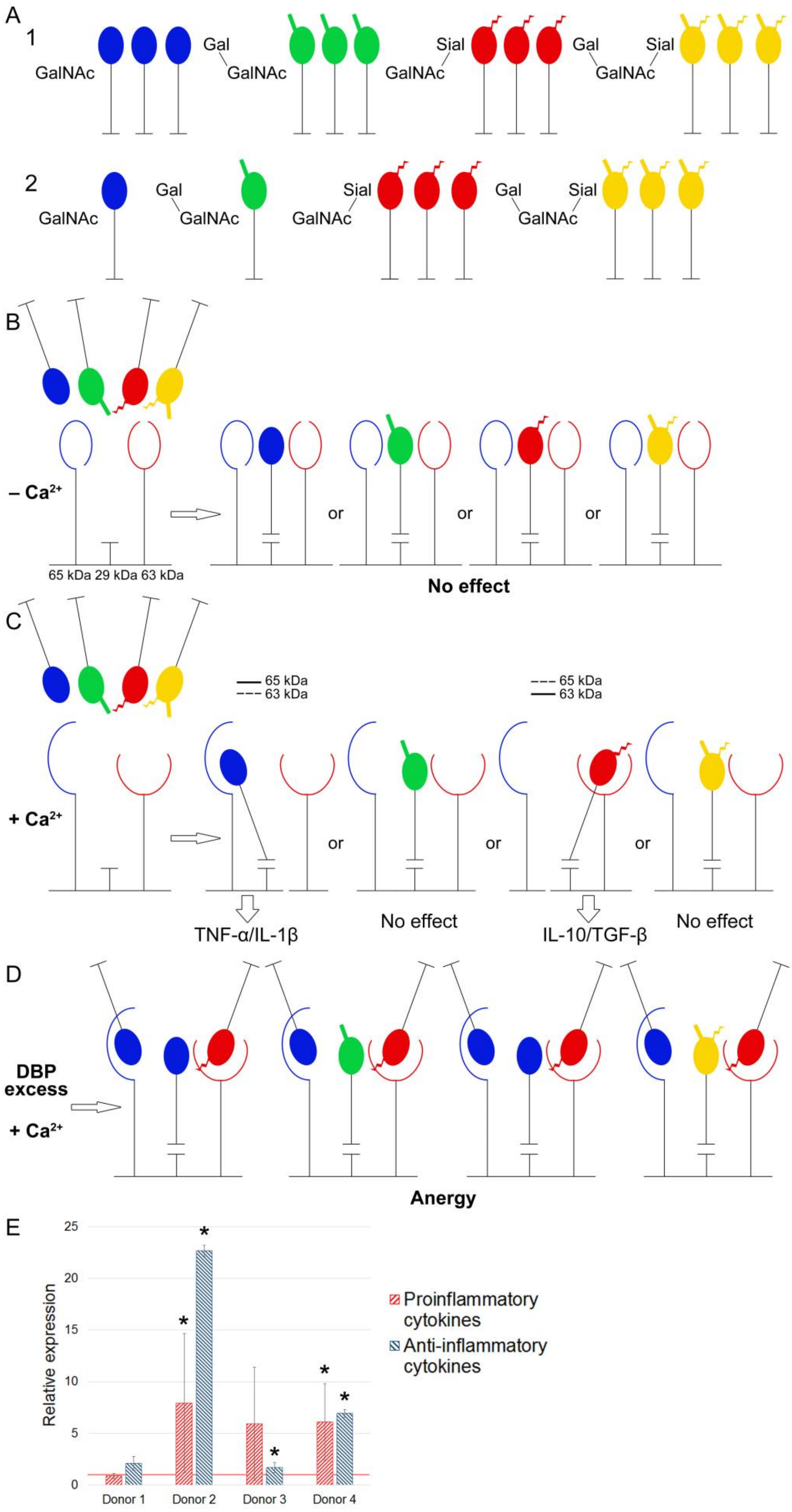
A
1
GalNAc
Gal
GalNAc
Sial
GalNAc
Gal
Sial
GalNAc
2
GalNAc
Gal
GalNAc
Sial
GalNAc
Gal
Sial
GalNAc
B
– Ca2+
65 kDa 29 kDa 63 kDa
or
or
or
No effect
C
65 kDa
63 kDa
65 kDa
63 kDa
+ Ca2+
or
or
or
TNF-α/IL-1β
No effect
IL-10/TGF-β
No effect
D
DBP excess
+ Ca2+
Anergy
E
Relative expression
0
5
10
15
20
25
Donor 1
Donor 2
Donor 3
Donor 4
Proinflammatory cytokines
Anti-inflammatory cytokines

Figure S2: A schematic view of the DBP structure and presumed variants of deglycosylation of GalNAc–sialic acid–galactose trisaccharide. A graphical representation of the structural (core) elements used to visualize the interaction between various glycosylated forms of DBP/GcMAF and the CLEC10A complex receptor is provided in Supplementary Figure 1S. (A) (1) Plasma contains equal amounts of all the possible variants of glycosylated DBP forms (the null hypothesis). (2) Variants of glycosylated DBP/GcMAF forms present in plasma with allowance for the available literature data (see explanation in the text). It is supposed that some DBP/GcMAF molecules carry GalNAc only (shown in blue GalNAc); some of them carry GalNAc + galactose (shown in green GalNAc-Gal); some of them carry GalNAc + sialic acid (shown in red GalNAc-Sial); or glycosylated molecules carrying all three sugar residues (GalNAc + galactose + sialic acid) are present (shown in yellow GalNAc-Gal-Sial). (B) CLEC10A is present on the surface of PMs of C57BL/6 mice as three derivatives interacting with anti-CLEC10A antibodies: the canonical 29 kDa protein and two high-molecular-weight 63/65 kDa derivatives, which are presumably aggregated into a single co-functioning complex. Apparently, after treating PMs with the ligand, the 29 kDa CLEC10A derivative acts as a primary "anchor" and immobilizes the DBP/GcMAF molecule on the plasma membrane regardless of whether Ca2+ molecules are present or not. Since CLEC10A can bind to a single DBP/GcMAF molecule, there is competition for binding to the primary "anchor". In the absence of Ca2+, this contact seemingly causes nonspecific stochastic synthesis of mRNA of analyzed cytokines. (C) Epitopes, specific Ca2+-dependent high-molecular-weight derivatives of the 63/65 kDa CLEC10A receptor, "open up" at sufficiently high Ca2+ concentrations. Depending on which DBP/GcMAF molecule was bound to the primary "anchor", its secondary binding to the 63 kDa CLEC10A or 65 kDa CLEC10A derivatives takes place. If this molecule carries a terminal GalNAc moiety (shown in blue), it secondarily binds to the 65 kDa CLEC10A. If there is the "GalNAc + sialic acid" glycosylation type, secondary binding to the 63 kDa derivative takes place (shown in red). In both cases, the activated complex is internalized into the cytoplasm. In the former and latter cases, mRNA synthesis of pro- and anti-inflammatory cytokines is induced, respectively. Secondary binding does not occur if the molecule carries GalNAc + galactose or GalNAc + galactose + sialic acid, thus resulting in anergy and absence of cytokine mRNA synthesis. (D) An excessive amount of the ligand the presence of Ca2+ ions cause competitive interactions between the 29 kDa "anchor" derivative and the 63/65 kDa high-molecular-weight derivatives. It is hypothesized that when a double complex comprising the "anchor" (29 kDa) and Ca2+-dependent high-molecular-weight derivatives (63/65 kDa) of CLEC10A receptor with DBP/GcMAF molecules cannot be formed, complexes are aggregated due to interaction of the termini of GcMAF molecules not immobilized at the 29 kDa anchor derivative [28]. Synthesis of both pro- and anti-inflammatory cytokines is inhibited because of the lack of proper conditions for a functional complex to be formed and internalized into the cytoplasm. (E) Quantification of mRNA expression of the genes encoding pro- and anti-inflammatory cytokines in murine peritoneal macrophages activated by different DBP derived from four donors, at the same dose (2 μg) per 5×105 PMs [28]. The mRNA expression level in PMs cultured in RPMI-1640 medium assumed to be equal to unity (red solid line) is used as a control. The statistically significant differences compared to the control are denoted as: * – $p < 0.05$, the Mann–Whitney U test.
